# IMGN853 Induces Autophagic Cell Death in Combination Therapy for Ovarian Cancer

**DOI:** 10.1158/2767-9764.CRC-24-0215

**Published:** 2025-03-28

**Authors:** Anca Chelariu-Raicu, Thanh Chung Vu, Sujanitha Umamaheswaran, Elaine Stur, Pahul Hanjra, Yunah Han, Min Hu, Jerome Lin, Barrett C. Lawson, Jinsong Liu, Anil K. Sood, Yunfei Wen

**Affiliations:** 1 Department of Gynecologic Oncology and Reproductive Medicine, The University of Texas MD Anderson Cancer Center, Houston, Texas.; 2 Department of Obstetrics and Gynecology, University Hospital, LMU Munich, Munich, Germany.; 3 German Cancer Consortium (DKTK), partner site Munich, German Cancer Research Center, Munich, Germany.; 4 The University of Texas MD Anderson Cancer Center UTHealth Graduate School of Biomedical Sciences, Houston, Texas.; 5 Y.H., Davidson College, Davidson, North Carolina.; 6 Department of Systems Biology, The University of Texas MD Anderson Cancer Center, Houston, Texas.; 7 Department of Pathology and Laboratory Medicine, The University of Texas MD Anderson Cancer Center, Houston, Texas.

## Abstract

Antibodies targeting folate receptor 1 (FOLR1) induce autophagic cell death in addition to antibody-dependent cytotoxicity, but the biological relevance of anti-FOLR1 antibody–induced autophagy for clinical applications remains unclear. In this study, we investigated the role of autophagic cell death triggered by IMGN853 (mirvetuximab soravtansine), an FOLR1-targeted antibody–drug conjugate, and explored potential combinations of IMGN853 with chemotherapeutic drugs used for ovarian cancer treatment. We discovered that FOLR1 was predominantly expressed in epithelial ovarian cancer cells, with similar expression levels observed in both primary ovarian tumors and metastatic omental tumors from patients with high-grade serous ovarian cancer (HGSC). Treatment with IMGN853 improved survival in mice bearing patient-derived xenografts of HGSC, enhanced autophagic flux, and induced cell death in HGSC cells. Additionally, it increased expression of the autophagy-related marker LC3B-II and cell death as marked by cleaved caspase-3, in a manner dependent on beclin-1, in HGSC models. Notably, combinations of IMGN853 with topotecan or the anti–VEGF-A antibody (B20) significantly reduced tumor growth compared with IMGN853 alone, whereas no significant effect was observed with olaparib. Our findings indicate that IMGN853 induces autophagic cell death, which contributes to its tumor-inhibiting effects. The identification of these effective combination therapies and the mechanisms behind FOLR1-mediated autophagic cell death could facilitate further clinical development of IMGN853.

**Significance:**

FOLR1 is heterogeneously overexpressed in epithelial ovarian cancer. We examined the combined effects of the anti-FOLR1 antibody–drug conjugate (IMGN853) with other drugs, including topotecan, anti–VEGF-A antibody, and olaparib. These findings could contribute to the continued development of IMGN853 in the treatment of ovarian cancer.

## Introduction

Given that folate receptor 1 (FOLR1) is selectively overexpressed in epithelial ovarian cancer cells, initial clinical trials of FOLR1-targeting agents explored the efficacy of farletuzumab (MORAb-003), an anti-FOLR1 antibody, and vintafolide, a conjugate of folic acid (FA) and the vinca alkaloid desacetylvinblastine hydrazide (1–3). MORAb-003 was shown to reduce tumor growth through autophagic cell death (4). However, neither MORAb-003 nor vintafolide was found to have sufficient clinical benefit to warrant further development (5). Subsequent therapeutic studies focused on combining the specificity of an anti-FOLR1 antibody with a cytotoxic agent through antibody–drug conjugates (ADC). ADCs allow for more precise delivery of cytotoxic agents to cancer cells by targeting membrane-associated antigens that are specifically expressed on cancer cells; ADCs can also be combined with other chemotherapeutic agents. The ADC mirvetuximab soravtansine (IMGN853) is currently used for ovarian cancer treatment. It links a humanized monoclonal antibody (mAb) that targets FOLR1 to maytansinoid, a cytotoxic agent that disrupts microtubule assembly and induces mitotic arrest (6). The initial phase III FORWARD I trial (NCT02631876), which enrolled 366 patients with FOLR1^+^ platinum-resistant epithelial ovarian cancer (PROC), showed no significant difference in progression-free survival durations between patients treated with IMGN853 and those treated with chemotherapy (paclitaxel, PEGylated liposomal doxorubicin, or topotecan; ref. 7). However, the scoring of FOLR1 expression used in this trial was underperformed; approximately 30% of the patients enrolled in the study were actually not eligible as they had FOLR1^low^ tumors (8). In November 2022, IMGN853 received accelerated approval from the FDA for adult patients with FOLR1^+^ PROC, fallopian tube cancer, or primary peritoneal cancer who had received 1 to 3 prior systemic treatment regimens (https://www.fda.gov/drugs/resources-information-approved-drugs/fda-grants-accelerated-approval-mirvetuximab-soravtansine-gynx-fra-positive-platinum-resistant).

IMGN853 has been investigated in several clinical trials since its approval. In the single-arm SORAYA trial (NCT04296890), which enrolled approximately 110 patients with PROC, IMGN853 elicited an overall response rate of 32.4% (95% confidence interval, 23.6%–42.2%) but only in a prespecified FOLR1^+^ subgroup (9). The phase III MIRASOL randomized trial (NCT04209855) compared patients with PROC treated with IMGN853 with those who received investigator’s choice of chemotherapy (10). The study found that patients with high FOLR1 expression who received IMGN853 had significantly longer progression-free survival (5.62 vs. 3.98 months, *P* < 0.0001) and overall survival (16.46 vs. 12.75 months, *P* < 0.0046). In addition, ongoing clinical trials are testing combination therapy with IMGN853 and bevacizumab in patients with PROC (GLORIOSA), and with IMGN853 and carboplatin in patients with platinum-sensitive ovarian cancer (420 Study; ref. 11). To enhance the clinical efficacy of IMGN853, further exploration of combination strategies is warranted, and optimal approaches remain to be determined. The goal of our study is to determine the efficacy of IMGN853 as monotherapy and the optimal combinations with drugs used for ovarian cancer treatment. We examined the role of IMGN853-induced autophagic cell death and identified rational combinations of IMGN853 with several drugs, including topotecan, the PARP inhibitor olaparib, and the anti–VEGF-A antibody B20 using an array of ovarian cancer models including high-grade serous ovarian cancer (HGSC). Our results could have important implications for the design of combination therapies comprising IMGN853 and other drugs for the treatment of ovarian cancer, as well as other types of FOLR1^+^ cancers.

## Materials and Methods

### Cell lines and reagents

We used multiple epithelial ovarian cancer cell lines [endometrioid ovarian cancer cells SKOV3 (RRID: CVCL_0532); IGROV1(RRID:CVCL_1304); A2780 (RRID: CVCL_0134); and HGSC cell lines OVCA432 (RRID:CVCL_3769) and OVCAR-8 (RRID: CVCL_1629; ref. 12] to represent the different subtypes of ovarian cancer in this study. All cell lines were purchased from ATCC and authenticated by DNA profiling using short tandem repeat analysis at MD Anderson Cancer Center’s Cytogenetics and Cell Authentication Core. Cells were cultured in RPMI 1640 medium (Thermo Fisher Scientific) supplemented with 10% FBS and 0.1% gentamicin. Upon reaching ∼90% confluence, cells were passaged using 0.25% trypsin-EDTA (Thermo Fisher Scientific). OVCA432 and OVCAR-8 cells, which have been shown to be representative models of HGSC (13), were transfected with Firefly luciferase Lentifect purified lentiviral particles (GeneCopoeia) according to the manufacturer’s standard protocol to generate luciferase-labeled cells (OVCA432-luc and OVCAR-8-luc) for use with the IVIS optical imaging system (PerkinElmer).

To test the take-rate of the OVCAR-8 cell line, female athymic nude mice were intraperitoneally injected with 4 × 10^6^ OVCAR-8 cells. Approximately 4 weeks after injection, tumors were collected and mechanically dissociated into a single-cell suspension, which was seeded into cell culture plates. The cells were then propagated for five or six passages. The procedure was repeated in a similar manner to generate the OVCAR-8 ip2 cell line used in the *in vivo* experiments (Figs. 5–7) in this study. All cell lines were routinely tested for *Mycoplasma* contamination using a PCR Universal Mycoplasma Detection Kit (#30-1012K; ATCC).

IMGN853 (ImmunoGen, Inc.) was reconstituted in normal saline to a stock solution of 25 mg/mL, and the lyophilized powder of hydroxychloroquine (HCQ) sulfate (#H0915; Sigma-Aldrich) was reconstituted in DMSO (#D2650; Sigma-Aldrich) to a stock solution of 10 mmol/L according to the manufacturer’s instructions. Formulation buffer (ImmunoGen, Inc.) served as a control for IMGN853. Alexa Fluor 488–folic acid (FA)–conjugated submicrogels were prepared in-house, based on a previous protocol (14). The Human Folic Acid (Competitive EIA) ELISA Kit was purchased from LSBio and performed according to the manufacturer’s instructions. Topotecan (#T2705; Sigma-Aldrich) was reconstituted in a 20 mg/mL stock solution and diluted in sterile water prior to injection. Olaparib (#763113-22-0; Tecoland) was reconstituted in a 10 mg/mL stock solution using 10% DMSO and 30% Kleptose HPB parenteral hydroxypropyl β-cyclodextrin (Sigma-Aldrich). Working solutions were freshly prepared daily and administered within 1 hour of mixing. Murine anti–VEGF-A mAb B20 (Genentech) was prepared according to the manufacturer’s instructions. Short hairpin RNAs against human beclin-1 were purchased from OriGene.

### Human specimens and patient-derived xenograft tissue processing

The samples were collected with patient’s written informed consent as appropriate per the Institutional Review Board (IRB) approval at The University of Texas MD Anderson Cancer Center, with samples distributed via the Institutional Tissue Bank. The processing of patient-derived xenografts (PDX) of ovarian cancer was described previously (15). Clinical data of the patients are summarized in Supplementary Table S1. The clinicopathologic and genomic characterization of patients with HGSC from whom the PDX models were developed is included in Supplementary Fig. S4A. The process for IHC analysis of FOLR1 expression in PDX tumors is included in Supplementary Fig. S4B.

### *In vivo* experiments

All *in vivo* study protocols were reviewed and approved by MD Anderson’s Institutional Animal Care and Use Committee and were performed in accordance with the relevant guidelines and regulations. Female NOD/SCID, SCID, and athymic nude mice were purchased from Taconic Biosciences. All mice were 6 to 10 weeks old at the time of cell injection or tumor implantation.

For the dose-finding experiment, OVCA432 cells were surgically introduced via intraovarian injection. Approximately 21 days after cell injection, when the bioluminescence signal had reached 5.0 × 10^3^ photons/seconds/cm^2^/sr, the mice were randomized to receive weekly treatment with up to three doses of control formulation buffer; single-agent IMGN853 at concentrations of 2.6 mg/kg (0.5 nmol/L), 5.2 mg/kg (1.0 nmol/L), or 10.4 mg/kg (2.0 nmol/L); or single-agent DM4 (Ch-KTI-s-SPDB-DM4, a nonbinding antibody that has the same linker and payload as IMGN853 and served as a negative control) at a concentration of 10.4 mg/kg (2.0 nmol/L). After treatment initiation, mice were closely monitored for adverse effects. After 3 weeks of treatment, all mice were euthanized by cervical dislocation. At necropsy, the tumor weight, number of metastatic nodules, volume of ascites present, and body weight were recorded.

For the survival experiments, PDX models developed from patients with PROC HGSC were provided by the Ovarian Cancer Moon Shot Program at MD Anderson. Tissues used for the experimental protocols in this study were approved by MD Anderson’s IRB. PDX tumors were established by implanting tumor tissues into donor NOD/SCID mice. Briefly, each mouse was anesthetized using 2.5% isoflurane, and oxygen was delivered through a chamber. Under anesthesia, the mouse was positioned face down, and the scapular region was prepared by removing the fur. The surgical area was sanitized with chlorhexidine and isopropanol, and a 5-mm incision was made approximately 5 mm to the left or right of the spine underneath the skin layer. Using forceps, a 1- to 1.5-cm-deep pocket was created on both sides of the body by separating the tissue under the skin. The PDX tissue was then inserted into the far end of the pocket. When tumor sizes reached approximately 2 cm^3^, the xenograft tumors were harvested, mechanically smashed, mixed with ascites fluid from the same donor animal, and surgically implanted into 20 recipient SCID mice via intraovarian injection (16). Three weeks after reimplantation, the mice were randomized to receive either IMGN853 (5.2 mg/kg) or the control formulation buffer once weekly for 3 months. Animal survival was recorded individually and represented using Kaplan–Meier survival curves.

For combinational therapeutic experiments, OVCAR-8-luc cells were cultured to 70% confluence, trypsinized, washed once with PBS, resuspended in ice-cold Hank’s balanced salt solution, and intraperitoneally injected into approximately 6-week-old athymic nude mice (4 × 10^6^ cells/mouse, 0.2 mL/mouse). Tumor establishment was confirmed using bioluminescence imaging (BLI); when the bioluminescence signal reached approximately 5.0 × 10^3^ photons/seconds/cm^2^/sr), mice were randomized to receive control (formulation buffer, ImmunoGen, Inc.), single-agent IMGN853 (5.2 mg/kg, intraperitoneally, 1×/week), single-agent olaparib (50 mg/kg in PBS, oral gavage, daily), single-agent topotecan (7.5 mg/kg, intravenously, 1×/week), single-agent B20 (5 mg/kg, intraperitoneally, 2×/week), or combination of IMGN853 with olaparib, topotecan, or B20. Each group initially consisted of 10 mice. In Fig. 6, two mice from the control group became moribund before receiving the fourth dose and were excluded from the final analysis. All remaining mice were euthanized via cervical dislocation at the end of the experiment. During necropsy, body weight, tumor weight, tumor distribution, number of tumor nodules, and ascites volume were recorded. Tissue specimens were collected for subsequent analysis, including snap-freezing for protein analysis, fixation in formalin for paraffin embedding, and embedding in optimal cutting temperature medium (Miles, Inc.) for frozen slide preparation.

### Single-cell analysis of FOLR1 expression in different tumor-cell types

Sample collection for single-cell analysis was approved by MD Anderson’s IRB. All the patients provided informed consent for the use of their samples. Single-cell analyses were performed as described previously (17). Briefly, three samples (T1, T6, and T6.1) from two primary tumors (T1 and T6) and four samples (OMT-1.1, OMT-1.2, OMT-3.1, and OMT-3.2) from two metastatic omental tumors (OMT-1 and OMT-3) were obtained from chemotherapy-naïve patients diagnosed with HGSC. T6 and T6.1, OMT-1.1, OMT-1.2, OMT-3.1, and OMT-3.2 were separated from the original tumors into CD45^−^ and CD45^+^ samples. Sample processing was performed immediately after the tissue collection. Approximately 2 g of tissue was minced and dissociated using the Accumax cell dissociation solution (STEMCELL Technologies Inc.). After dissociation, the cells were washed with complete medium to stop the reaction, centrifuged, resuspended in freezing medium (90% FBS/10% DMSO), and frozen at −80°C. For single-cell analysis, the cells were thawed, stained with LIVE/DEAD Aqua (Thermo Fisher Scientific) and an anti-CD45 antibody, and sorted using a BD FACSAria flow cytometer. Sorted cells were processed using Chromium Single Cell 3′ Reagent Kits (v3 Chemistry, 10× Genomics).

Single-cell data were submitted to Gene Expression Omnibus under accession number GSE181955. The data were analyzed using Cell Ranger software version 3.1.0, and a gene expression matrix based on universal molecular identifier counts was generated. We employed Seurat version 3.1.4 for unbiased cell clustering analysis. Briefly, we first filtered the matrix based on minimum and maximum cutoffs for genes/cell, cells/gene, and optional parameters such as mitochondrial gene universal molecular identifier count as a percentage of the total, and then normalized the data. Next, variable genes were identified, and the data were scaled. We then identified principal components and, finally, identified cell “neighbors” and cell clusters all using the Seurat pipeline.

### IHC and immunofluorescence staining

Sample collection for IHC analysis was approved by the MD Anderson’s IRB. Formalin-fixed, paraffin-embedded samples from primary tumor tissues of patients with FOLR1^+^ HGSC were sectioned at 5 μm for IHC staining with FOLR1 antibody to determine the expression of FOLR1. All sections were reviewed by a board-certified pathologist who was blinded to the patients’ clinical outcomes.

Snap-frozen sections of tumor tissues from mice were fixed in cold acetone and treated with fish gelatin (Aurion) to block antibody binding of nonspecific epitopes. Next, the sections were incubated with primary antibodies against FOLR1, LC3B, cleaved caspase-3, hypoxia-inducible factor-1α (HIF1α), beclin-1, or E-cadherin (Supplementary Table S2) and then incubated with a fluorescence-conjugated secondary antibody. The sections were then washed with PBS, counterstained with Hoechst for 5 minutes, and mounted with Antifade Mountant (Thermo Fisher Scientific). Representative images were selected from three random fields at 20X objective magnification using a confocal microscope (Nikon). Terminal deoxynucleotidyl transferase–mediated dUTP nick end labeling assay was used to detect apoptotic cells in HGSC PDX tumor tissues according to the manufacturer’s protocol (Thermo Fisher Scientific).

### Acridine orange and SYTOX blue staining and flow cytometry

To characterize autophagic flux, we performed acridine orange staining to detect acidic vesicular organelles (AVO). A2780, OVCA432, and OVCAR-8 cells were seeded in six-well plates at a density of 3 × 10^4^ cells/well in triplicate. After 24 hours, IMGN853 (1.0 nmol/L), HCQ sulfate (400 nmol/L), or IMGN853+HCQ was added to the medium. After 72 hours, acridine orange stain was mixed and added to the media in the dark at a concentration of 1 ng/mL, and the cultured cells were then incubated for at least 30 minutes. Cells were then harvested under EDTA-free conditions, and the formation of AVOs was visualized using an LSRFortessa X-20 cell analyzer. The ratio of red fluorescence at 600 nm to green fluorescence at <500 nm (emission wavelength) was plotted. To detect cells with damaged cell membranes (i.e., dead cells), we performed parallel experiments in which the cells were harvested after 72 hours of treatment and subjected to SYTOX blue staining. SYTOX^+^ cells were identified using an LSRFortessa X-20 cell analyzer and gated by nonstaining controls.

### MTT cell viability assay

OVCAR-8 cells were seeded in 96-well plates at an initial density of 3 × 10^3^ cells/well in quadruplicate. After 24 hours, culture medium with IMGN853, topotecan, or both at escalating concentrations (0.01, 0.1, 0.5, 1.0, 5.0, and 10.0 nmol/L) was added. Culture medium without drug was used as a control. After 72 hours of incubation, 50 μL of 1.5 mg/mL 3-(4,5-Dimethylthiazol-2-yl)-2,5-Diphenyltetrazolium Bromide (MTT) (#J19265; Affymetrix) solution was added to each well, and the plates were incubated for an additional 2 hours. The medium containing MTT was discarded, 100 mL of DMSO was added to each well for completely re-suspending the MTT byproduct. The plates were analyzed using a spectrophotometer plate reader at an absorbance of 570 nm. To calculate the percentage of viable cells, the mean absorbance values for each treatment group were normalized to those of the cells treated with culture medium. Potential synergism between IMGN853 and topotecan or olaparib was assessed using median effect analysis, and the combination indices (CI) were calculated and reported as the combination ratio of CI/fraction affected (Fa; default effect) using CompuSyn software (18).

### Confocal microscopy and fluorescence intensity quantification

The stained cells were visualized using a Nikon laser scanning confocal microscope with a 20× long working distance lens. Each experiment was repeated at least 2 times, and representative images are shown. Fluorescence intensity was quantified using the cell function in Imaris Cell 9.0. The sum of fluorescence intensity (relative fluorescence unit) in each channel was plotted according to Imaris Reference Manual (Oxford). The results were obtained from *n* = 3 individual repeats but not statistically compared.

For real-time imaging, OVCAR-8 cells expressing a tandem monomeric pEGFP-RFP–tagged LC3 plasmid (*ptfLC3–pEGFP-RFP*) were treated with control formulation buffer or IMGN853 for 48 hours and then imaged for 48 to 72 hours in an incubator chamber at 37°C with 5% CO_2_ using a Leica confocal microscope at 30-minute intervals. Images were processed using Leica Application Suite X software.

### Transmission electron microscopy

Samples were fixed with a solution containing 3% glutaraldehyde plus 2% paraformaldehyde in 0.1 mol/L cacodylate buffer, pH 7.3, then washed in 0.1 mol/L sodium cacodylate buffer and treated with 0.1% Millipore-filtered cacodylate-buffered tannic acid, postfixed with 1% cacodylate-buffered osmium tetroxide; and stained *en bloc* with 1% Millipore-filtered aqueous uranyl acetate. The samples were dehydrated in increasing concentrations of ethanol, infiltrated, and embedded in LX-112 medium. The samples were polymerized in an oven at 60°C for 3 days. Ultrathin sections were cut in a Leica Ultracut microtome, placed on formvar-coated single-slot copper grids, stained with uranyl acetate and lead citrate, and examined in a JEM 1010 transmission electron microscope (JEOL USA, Inc.) at an accelerating voltage of 80 kV. Digital images were obtained using an AMT Imaging System (Advanced Microscopy Techniques Corp.).

### Statistical analysis

For the *in vivo* therapy experiments, 10 mice were assigned to each treatment group at the first dose. Using an ANOVA model with 10 mice per arm, an effect size of 0.45 could be detected with 80% power. At the conclusion of the experiments, mean tumor and body weights were compared across treatment groups, with statistical significance set at *P* ≤ 0.05. All statistical tests were two-sided and performed using GraphPad Prism 7.0 (RRID: SCR_002798). For the PDX 2414 survival experiment in Fig. 2C, Kaplan–Meier survival curves were generated using R version 3.4.1 and analyzed with log-rank tests. For the PDX 2428 cisplatin-resistant model in Supplementary Fig. S4D, the results were assessed using 2×2 ANOVA models for continuous endpoints and Fisher exact tests for binary outcomes. As the *in vivo* dose-finding experiment in Supplementary Fig. S3 was designed to find the optimal dose of IMGN853, only three mice were assigned per treatment group.

The results of the *in vitro* experiments, including flow cytometric analyses of AVOs and dead cells, were analyzed and compared using GraphPad Prism 8.0. The groups were compared using the Student *t* test, and *P* values ≤ 0.05 were considered statistically significant. For all *in vitro* assays, measurements are presented as the mean ± SEM, unless specifically noted otherwise.

### Data availability

Single-cell RNA sequencing data are available in the Gene Expression Omnibus using accession number GSE181955. All other data access requests will be considered by a Data Access Committee. All reagents and materials in this study are available upon request and upon completion of a Materials Transfer Agreement from the corresponding author at ywen2@mdanderson.org.

## Results

### Heterogeneity of FOLR1 expression in HGSC tumors

**Figure 1:**
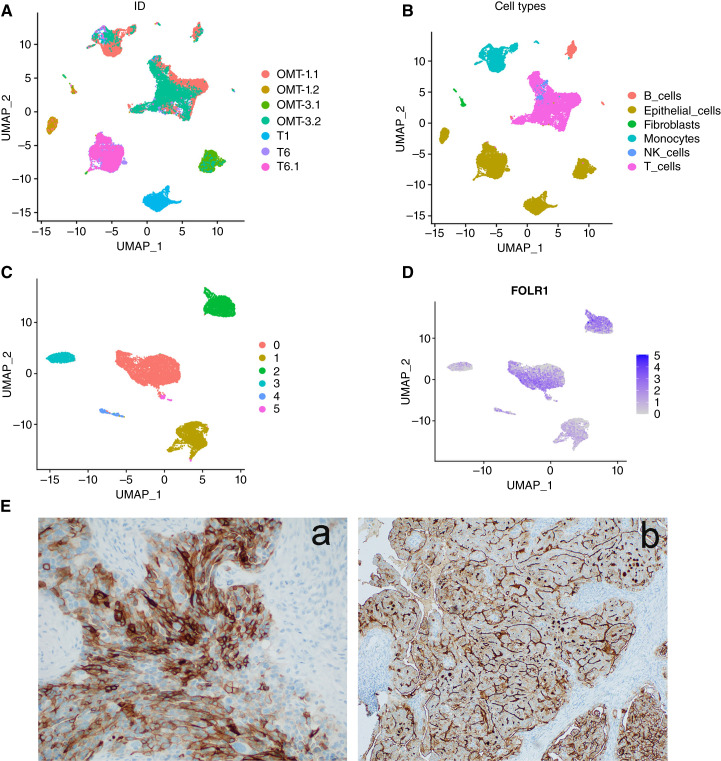
Ovarian high-grade serous carcinomas (HGSCs) have heterogeneous FOLR1 expression. **A** and **B,** UMAP visualization of major cell types in five freshly resected tumors of patients with HGSC [three primary tumors (T) and two omental metastases (OMT)] by sample type (**A**) and cell type (**B**). **C** and **D,** UMAP visualization of FOLR1 expression in epithelial cells after reclustering of the cells by the level of FOLR1 expression. **E,** Representative IHC images in tissue sections of primary tumors from two patients with HGSC showing epithelial expression of FOLR1 (a: chemotherapy-naïve HGSC; b: HGSC treated with neoadjuvant chemotherapy). UMAP, Uniform Manifold Approximation and Projection for Dimension Reduction.

Accurate quantification of FOLR1 expression in tumor cells is important for predicting the efficacy of FOLR1-targeting therapies. To assess the heterogeneity of FOLR1 expression in HGSC tumors, we performed single-cell analyses on primary tumors (T1, T6, and T6.1) and metastatic omental tumors (OMT-1.1, OMT-1.2, OMT-3.1, and OMT-3.2) from chemotherapy-naïve patients diagnosed with advanced ovarian HGSC (19). First, we performed sample clustering to identify the major cell types present, such as T cells, monocytes, epithelial cells, fibroblasts, NK cells, and B cells (Fig. 1A and B). Subsequently, we examined FOLR1 expression levels across these clusters and specific cell types, revealing that epithelial cells exhibited the highest levels of FOLR1 expression (Supplementary Fig. S1A and S1B). We further defined FOLR1 expression levels within the six epithelial cell clusters (Fig. 1C and D). Notably, primary HGSC tumor cells and metastatic omental epithelial cells demonstrated comparable FOLR1 expression levels (Supplementary Fig. S1C and S1D). To validate these findings, we conducted IHC staining for FOLR1 on HGSC tumors from two patients—one chemotherapy-naïve and one who had received neoadjuvant chemotherapy. The results confirmed that FOLR1 is predominantly expressed in epithelial cancer cells, with minimal expression in stroma (Fig. 1E). We further evaluated FOLR1 expression in 40 additional ovarian cancer samples with various histologic subtypes (20 HGSC, 10 endometrioid, and 10 clear-cell cases) from patients undergoing treatment with IMGN853. FOLR1 expression was scored using the VENTANA FOLR1 (FOLR1-2.1) RxDx Assay, which indicated that the majority of FOLR1 localization occurred in the epithelial tumor cells rather than stromal regions (Supplementary Table S1).

### IMGN853 decreases FA uptake in ovarian cancer cells

To evaluate the impact of IMGN853 on FA uptake in ovarian cancer cells, we used Alexa Fluor 488–FA–conjugated submicrogels in SKOV3 cells, which are known for their high FOLR1 expression (4). We compared FA uptake between cells with or without pretreatment with IMGN853 for 2 or 16 hours. Alexa Fluor 488–FA uptake was reduced after 2 hours of IMGN853 pretreatment and was nearly nonexistent after 16 hours (Supplementary Fig. S2A). The FA uptake was also measured in an HGSC cell line, OVCAR-8, by an intracellular FA ELISA. IMGN853 treatment decreased the intracellular levels of FA in OVCAR-8 cells over a longer period of time (12–48 hours; Supplementary Fig. S2B). These findings demonstrated that IMGN853 treatment decreased FA intake in ovarian cancer cells.

We queried the RNA sequencing dataset in the Cancer Cell Line Encyclopedia (https://depmap.org/portal/gene/FOLR1; ref. 20) to examine the FOLR1 expression in common HGSC cell lines (21) as well as some non-HGSC cell lines; high expression of FOLR1 was noted in most HGSC cell lines in comparison with the A2780 cell line, which was used as a FORL1^low^ control (Supplementary Table S3; refs. 20, 22). Protein expression analysis using an anti–human FOLR1 antibody further validated the presence of FOLR1 in OVCAR-8 and OVCA432 cells, with levels comparable to those in IGROV1 and SKOV3 cells, which are known as FOLR1^+^cell lines (4). On the other hand, the A2780 cell line was confirmed as FOLR1^low^ cells (Supplementary Fig. S3A).

### IMGN853 decreases FOLR1^+^ HGSC tumor growth and prolongs survival in platinum-resistant HGSC PDX models

Next, we established the optimal *in vivo* dose of IMGN853 using an orthotopic OVCA432 mouse model (Supplementary Fig. S3B). A weekly dose of 5.2 mg/kg (1.0 nmol/L) was selected for further experiments, as no additional benefits were observed at higher doses in reducing tumor weight (Supplementary Fig. S3C), the number of metastatic nodules (Supplementary Fig. S3D), or the volume of ascites collected from the peritoneal cavity (Supplementary Fig. S3E). IMGN853 was well tolerated across doses ranging from 2.6 to 10.4 mg/kg, as reflected by -the stable body weights of the mice at the end of the experiments (Supplementary Fig. S3F). IMGN853 treatment significantly decreased the percentage of metastatic nodules across most sites, including the peritoneum, pelvic sidewall, omentum, diaphragm, pelvis, spleen, kidney, and porta hepatis (Supplementary Fig. S3G). Notably, the percentage of mesenteric metastases in the 1.0 nmol/L IMGN853 group was slightly higher than that in the 0.5 nmol/L group, though not significantly (*P* = 0.5911), possibly due to variations in surgical tumor-cell implantation. Tumor sections were analyzed for expression of cleaved caspase-3 as a cell death marker (Supplementary Fig. S3H). Significantly higher cleaved caspase-3 positivity (*P* < 0.001) was observed in tumors treated with 5.2 mg/kg (1.0 nmol/L) IMGN853 compared with controls (Supplementary Fig. S3H).

**Figure 2:**
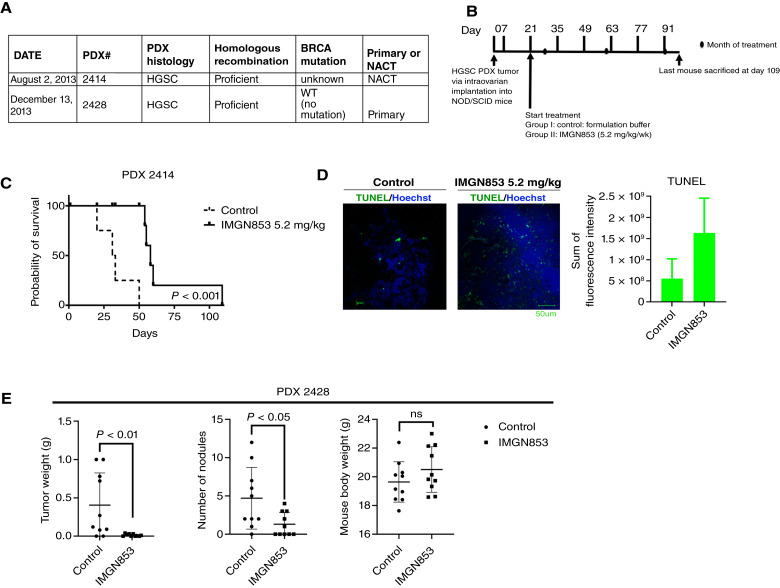
Effects of IMGN853 on the survival and inhibition of tumor growth of mice bearing ovarian high-grade serous carcinoma (HGSC) PDX tumors. **A,** Main clinical and pathologic characteristics of PDX tumor tissues from patients with HGSC. Date of Implantation (DATE) **B,** Schematic representation of PDX experiments. PDX mouse models were established by surgical implantation of HGSC tumors via intraovarian injection. Treatment with either formulation buffer (control) or IMGN853 (5.2 mg/kg, once weekly) was initiated approximately 3 weeks following tumor implantation in the recipient NOD/SCID mice. Black dots represent 1, 2, and 3 months, respectively, after PDX tumor implantation. **C,** Kaplan–Meier survival curves for HGSC PDX 2414–bearing mice treated with formulation buffer (blue) or IMGN853 (red). *P* < 0.001, log-rank test. **D,** Left, Representative confocal IF images showing terminal deoxynucleotidyl transferase–mediated dUTP nick end labeling (TUNEL) staining (green) in the control formulation buffer and IMGN853 treatment groups. Right, The fluorescence intensity from the green channel over that of the blue channel was quantified using Imaris v. 9.1.2. The sum of fluorescence intensity is shown in relative fluorescence unit (RFU). **E,** The HGSC PDX model 2428 was surgically implanted in NOD/SCID mice. Twenty-one days after implantation, mice were randomized to receive buffer (control) or IMGN853 (5.2 mg/kg, once weekly) for 6-dose treatment. Tumor weights (left), numbers of nodules (middle), and body weights (right) of the mice were recorded at necropsy. *P* values were determined by a two-tailed, nonparametric *t* test. Error bars ± SDs. NACT, neoadjuvant chemotherapy; WT, wild-type.

Considering the significance of PDX models in evaluating targeted therapies for ovarian cancer (23), we evaluated IMGN853’s effects on a set of orthotopic HGSC PDX models derived from patients with platinum-resistant HGSC (PDX 2414 and PDX 2428; Fig. 2A). Both PDX tumors express ∼100% positivity of FOLR1 according to their IHC staining (Supplementary Fig. S4A and S4B). Additionally, cisplatin resistance of these PDX tumors was confirmed *ex vivo* and *in vivo* (Supplementary Fig. S4C and S4D). First, we performed a survival experiment in HGSC PDX 2414 model using NOD/SCID mice (Fig. 2B). Kaplan–Meier survival analysis indicated that IMGN853-treated mice had significantly longer survival than buffer-treated controls (*P* < 0.001; Fig. 2C). Additionally, a terminal deoxynucleotidyl transferase–mediated dUTP nick end labeling assay revealed a higher proportion of apoptotic cells in IMGN853-treated tumors in comparison with the control group (Fig. 2D). Next, we performed a therapeutic experiment in NOD/SCID mice carrying orthotopic HGSC PDX 2428 tumor. After treatment with six doses, IMGN853 significantly reduced tumor weights (*P* < 0.01) and resulted in fewer tumor nodules (*P* < 0.05) compared with those treated with control agent alone [Fig. 2E (left and middle)]. As expected, IMGN853 alone was well tolerated by mice, as reflected by their stable body weights at the end of the experiment [Fig. 2E (right)]. Taken together, these findings demonstrate that IMGN853 treatment enhances survival and reduces tumor burden in animals with platinum-resistant HGSC PDX tumors.

### IMGN853 induces autophagic cell death in HGSC cell lines

**Figure 3:**
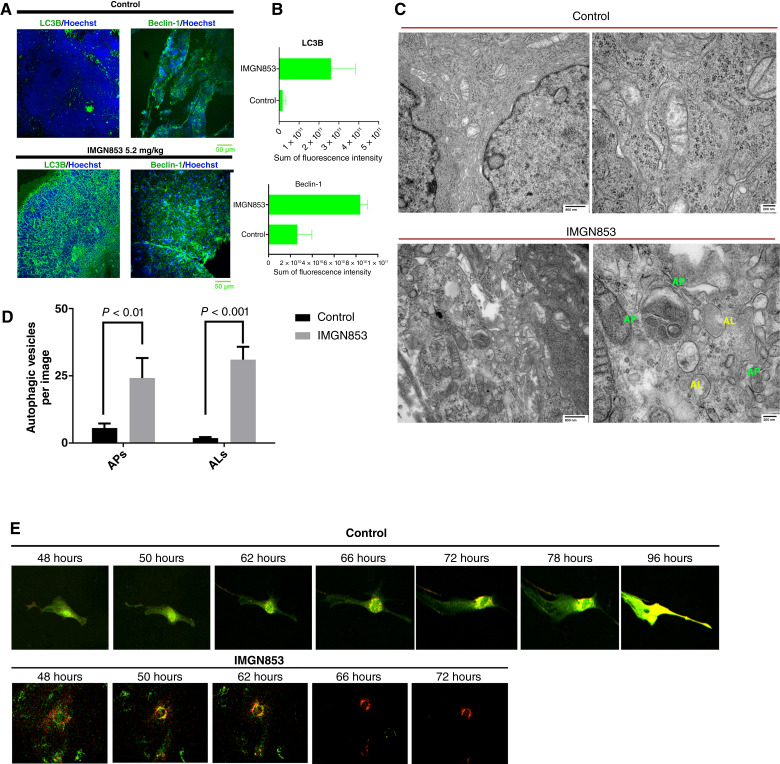
IMGN853 treatment increases autophagic flux and cell death in FOLR1^+^ ovarian high-grade serous carcinoma (HGSC) models. **A,** Representative confocal IF images showing expression patterns of the autophagy markers LC3B (green) and beclin-1 (green) in the control- (formulation buffer, top row) and IMGN853-treated PDX tumors (bottom row). **B,** Fluorescence intensity in each IF image was quantified using Imaris v. 9.1.2. The sum of fluorescence intensity for each marker is shown in relative fluorescent units (RFU); tissues treated with IMGN853 showed higher expression of LC3 and beclin-1. **C,** Representative images from transmission electron microscopy showed autophagic vesicles formed in the orthotopic OVCAR-8 tumors treated with control agent (top) or IMGN853 (bottom). Substantial amounts of APs (green) and ALs (yellow) were identified in the IMGN853-treated OVCAR-8 tumor cells, whereas tight cell–cell junctions and condensed patterns of mitochondria were observed in control tumors, in which only the minimum amounts of AP present. Substructure was observed at 20,000× (left) and 50,000× (right) magnifications. Representative ultrathin sections in representative areas from *n* = 5 fields are shown. **D,** Quantification of autophagic vacuoles including APs and ALs per image at 20,000× magnification (approximately 44 μm^2^). *P* values were determined by a two-tailed, nonparametric *t* test. Error bars ± SDs. **E,** ptfLC3–pEGFP-RFP–expressing OVCAR-8 cells were treated with control (top) or IMGN853 (bottom) for 48 hours and imaged using a Leica confocal microscope for another 24–48 hours. Time-lapse videos were recorded to assess autophagy flux (represented by GFP-LC3 and RFP-LC3 colocalization during cell division) under the control condition or the accumulation of late-stage ALs (represented by RFP-LC3) leading to autophagic cell death under the IMGN853 condition (available as Supplementary Movies S1 and S2). The time points are shown above each image, *n* = 3 for each treatment.

To understand the role of autophagic cell death in the therapeutic effects of IMGN853, we assessed levels of the autophagosome (AP) marker LC3B and the autophagy regulator beclin-1 in both control and IMGN853-treated PDX tumors. IMGN853 treatment markedly increased both LC3B and beclin-1 expression (Fig. 3A and B). We next explored the effect of IMGN853-induced autophagy on FOLR1^+^ OVCAR-8 tumors. OVCAR-8 tumors were first examined with transmission electron microscopy. We observed that the control tumors had the typical ultrastructural features of a neoplasm, including tight cell–cell junctions and condensed patterns of mitochondria [Fig. 3C (Top)]. However, in the IMGN853-treated OVCAR-8 tumors, there were large numbers of APs (green) bordered by double membranes, as well as single membrane–bound autolysosomes [AL, yellow; Fig. 3C (Bottom)]. Detailed fusion of APs with lysosomes, and lipid droplets into ALs was also observed in the cytoplasmic area of IMGN853-treated OVCAR-8 tumor cells [Fig. 3C (bottom right)]. Significant increases in autophagic vacuoles including APs and ALs were observed in IMGN853-treated OVCAR-8 tumors in comparison with control tumors (Fig. 3D). For higher resolution images, please refer to Supplementary Fig. S5.

To further validate these findings, we conducted time-lapse microscopy to monitor autophagic flux in IMGN853-treated OVCAR-8 cells. Before treatment, a ptfLC3–pEGFP-RFP construct was stably expressed in these cells. The acid-sensitive mGFP protein degrades upon AP–lysosome fusion, whereas the mRFP protein remains stable (24). Autophagic punctum expressing GFP+RFP+ LC3 persisted in control cells 48 to 72 hours after treatment, with cells completing mitosis (Fig. 3E, top row, and Supplementary Movie S1). Conversely, IMGN853-treated cells exhibited substantial RFP+LC3 in the cytosol, indicative of late-stage ALs, and cell death occurred 72 hours after treatment (Fig. 3E, bottom row and Supplementary Movie S2). Collectively, these results suggest that IMGN853 induces late-stage autophagy in FOLR1^+^ ovarian cancer cells, which contributes to its inhibitory effects on HGSC tumor cells.

**Figure 4:**
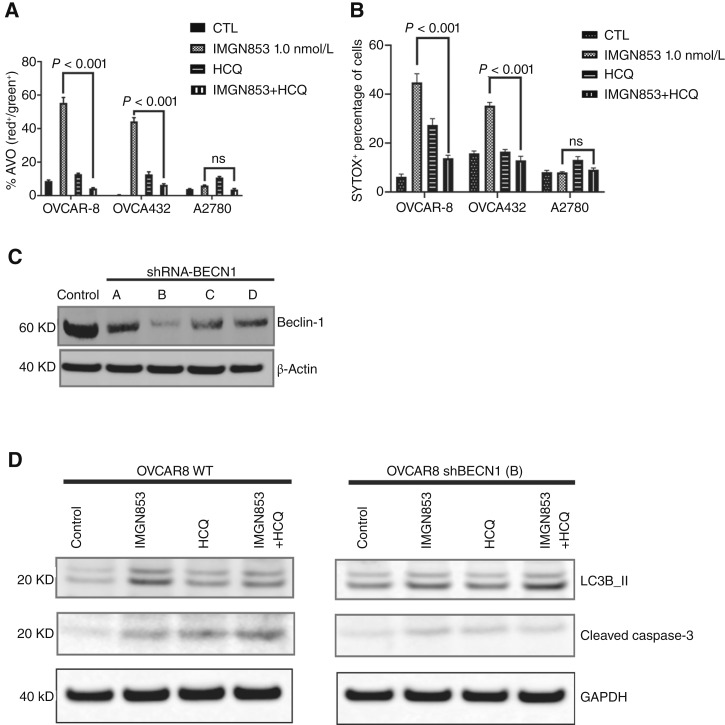
IMGN853 induces beclin-1–dependent autophagic cell death in FOLR1^+^ HGSC cell lines. **A,** Percentages of autophagic vacuoles in FOLR1^+^ OVCAR-8 and OVCA432 cells and in FOLR1^low^A2780 cells treated with control (formulation buffer), IMGN853 alone, HCQ alone, or IMGN853+HCQ for 48 hours and analyzed by acridine orange staining followed by FACS. *n* = 6; error bars represent SEMs. **B,** Percentages of SYTOX^+^ dead cells among FOLR1^+^ OVCAR-8 and OVCA432 cells and FOLR1^low^ A2780 cells treated with control (formulation buffer), IMGN853 alone, HCQ alone, or IMGN853+HCQ for 48 hours and analyzed by FACS. *n* = 6; error bars represent SEMs. **C,** Stable knockdown of beclin-1(*BECN1*) human shRNAs (A, B, C, and D, OriGene) were performed in OVCAR-8 cells. β-Actin was applied as the loading control. shRNA-B is selected for the subsequent experiments. The blots are representative of two biological repeats. shRNA, short hairpin RNA. **D,** Levels of LC3B-II and cleaved caspase-3 in OVCAR-8 cells WT (left) and shBECN1 knockdown (right) treated with control, IMGN853 alone, HCQ alone, or IMGN853+HCQ for 48 hours. β-Actin was used as the loading control. Each western blot was representative of two biological repeats. CTL, control, WT, wild-type.

To elucidate the mechanisms behind the autophagy-driven effects of IMGN853, we investigated autophagy pathway activities in FOLR1^+^ OVCAR-8, FOLR1^+^ OVCA432, and FOLR1^low^ A2780 cells *in vitro*. Autophagic flux is typically initiated by the formation of double-membraned AVOs (or APs), which are detectable using dual-color dyes like acridine orange (25). We used this method to quantify AVO accumulation within the cytoplasm of OVCAR-8, OVCA432, and A2780 cells. Acridine orange becomes protonated and trapped within membrane-bound organelles, emitting bright red fluorescence in high-pH compartments such as ALs, whereas it fluoresces bright green in neutral or low-pH environments such as early-stage APs (25). To assess the role of autophagic flux in IMGN853-induced cell death, we applied HCQ, a late-stage autophagy inhibitor that disrupts autophagic vacuolization and fusion (26). We measured the red/green fluorescence signal ratio emitted from acridine orange to indicate AVO accumulation under different treatments. IMGN853 combined with HCQ significantly reduced accumulation of AVOs in both OVCAR-8 and OVCA432 cells compared with IMGN853 treatment alone (*P* < 0.001) but showed no effect on FOLR1^low^A2780 cells (Fig. 4A). This reduction likely stems from HCQ’s inhibition of lysosomal acidification, blocking the IMGN853-induced accumulation of late-stage ALs. SYTOX staining and flow cytometry indicated that compared with IMGN853 alone, IMGN853 combined with HCQ led to significantly less cell death in FOLR1^+^ OVCA432 and OVCAR-8 cells (*P* < 0.001) but not in FOLR1^low^ A2780 cells (Fig. 4B).

Next, we analyzed the roles of the autophagy regulator beclin-1, the autophagy flux marker LC3B-II, and the cell death marker cleaved caspase-3 in OVCAR-8 cells treated with IMGN853 or HCQ, either alone or in combination. We used short hairpin RNAs against beclin-1 to knock down its endogenous levels in OVCAR-8 cells and selected shBECN1 (B) (Fig. 4C). We found that the levels of LC3B-II were elevated by IMGN853 or IMGN853+HCQ treatment only in the OVCAR-8 wild-type cells. Beclin-1 knockdown reversed the increased LC3B-II by IMGN853 treatment. In parallel, the levels of cleaved caspase-3 were higher in IMGN853- or IMGN853+HCQ-treated OVCAR-8 wild-type cells in comparison with control; however, the levels of cleaved caspase-3 remained the same across groups treated with control, IMGN853, HCQ, or IMGN853+HCQ in OVCAR-8 shBECN1 cells (Fig. 4D). Therefore, our data showed that IMGN853 induced autophagic cell death in the HGSC OVCAR-8 cell line in a beclin-1–dependent manner.

### Combinational therapies with IMGN853 in orthotopic FOLR1^+^ HGSC models

**Figure 5:**
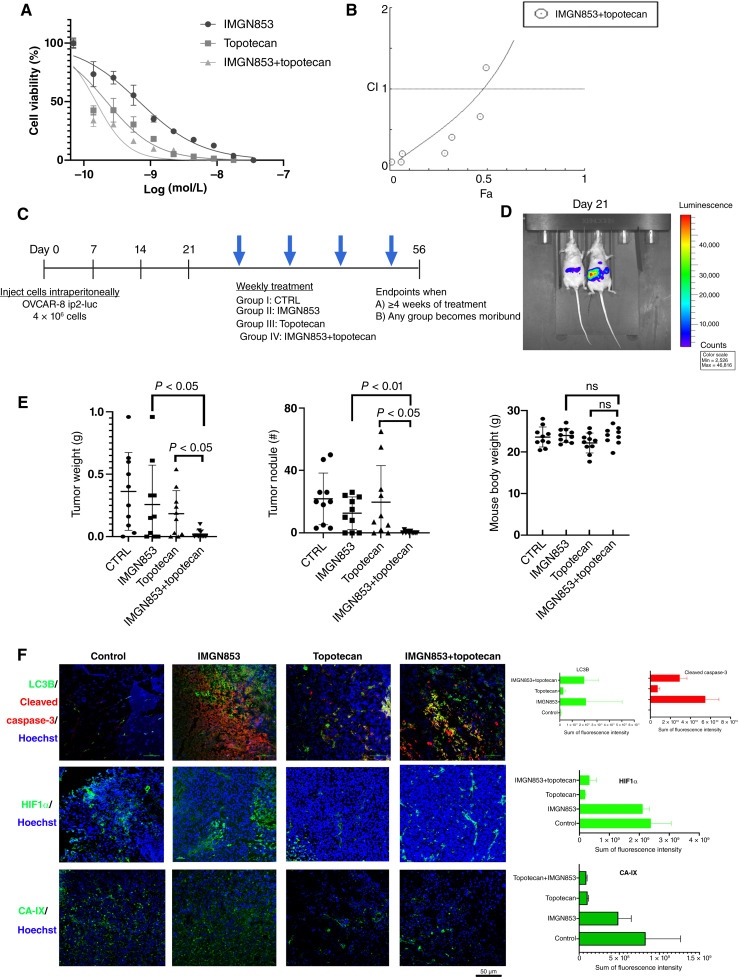
Combination therapy with IMGN853 and topotecan in the OVCAR-8 model. **A,** Results of MTT assays showing the combinatorial effects of different concentrations of IMGN853 with topotecan on the viability of OVCAR-8 cells after 72 hours of treatment [shown as log IMGN853(M)]. *n* = 3; data are mean ± SD. **B,** Synergy quantification of drug combination using Chou–Talalay analysis was reflected by the CI plot for IMGN853 and topotecan. **C,** Schematic representation of the mouse models of ovarian high-grade serous carcinoma (HGSC) established using luciferase-labeled OVCAR-8 ip2 cells (OVCAR-8 ip2-luc, 4 × 10^6^/mouse). **D,** Twenty-one days after cell injection as shown by bioluminescence signals in the representative mice from IVIS imaging, mice were randomized to receive buffer (control), IMGN853 (5.2 mg/kg, once weekly), topotecan (5 mg/kg, once weekly), or IMGN853+topotecan. **E,** Tumor weights (left), numbers of nodules (middle), and body weights (right) of the mice at necropsy. *P* values were determined by a two-tailed, nonparametric *t* test. Error bars ± SDs. **F,** Left, Representative IF images showing the expression of the autophagy marker LC3B, the cell death marker cleaved caspase-3, HIF1α, and the hypoxia markers CA-IX in tumor sections from each treatment group. Scale bar, 50 μm. Right, The fluorescence intensity of LC3B (green channel) and cleaved caspase-3 (red channel), HIF1α, or CA-IX over that of Hoechst (blue channel) in each image was quantified using Imaris v. 9.1.2. The sum of fluorescence intensity is shown in relative fluorescent units (RFU; *n* = 3 ± SDs). CTRL, control; ns, not significant.

To explore effective combination therapies with IMGN853, we initially selected topotecan, a topoisomerase I inhibitor that is clinically used for the treatment of recurrent PROC (27). *In vitro* studies of the combination of IMGN853 and topotecan in OVCAR-8 cells demonstrated a synergistic interaction, as evidenced by lower IC_50_ values for the combination treatment compared with each drug alone (Fig. 5A). The CI (CI/Fa) for IMGN853 and topotecan was 0.79/0.4 (Fig. 5B).

For *in vivo* studies, we injected luciferase-labeled OVCAR-8 ip2-luc cells into the peritoneal cavities of female athymic nude mice. Following tumor establishment confirmed through BLI (Fig. 5D), mice were randomly allocated to receive IMGN853, topotecan, the combination of IMGN853 and topotecan, or a control formulation buffer for 4-week doses (Fig. 5C). Mice treated with the combination therapy exhibited significantly lower tumor weights (IMGN853 vs. combination *P* < 0.05 and topotecan vs. combination *P* < 0.05) and fewer tumor nodules (IMGN853 vs. combination *P* < 0.01 and topotecan vs. combination *P* < 0.05; Fig. 5E). Each group including IMGN853 alone or in combination with topotecan showed stable body weights at the end of the experiment (Fig. 5E). Immunofluorescence (IF) and fluorescence intensity analyses revealed that tumors treated solely with IMGN853 or in combination with topotecan resulted in markedly higher LC3B and cleaved caspase-3 levels in comparison with those treated with control or topotecan only, correlating with increased autophagic cell death (Fig. 5F). As topotecan functionally inhibits HIF1α (28), treatment with topotecan alone or combined with IMGN853 resulted in reduced HIF1α levels in tumors (Fig. 5F). In concordance with the effects on HIF1α, we further used CA-IX to compare the hypoxia condition in the resulting tumors and found that both topotecan alone or combined with IMGN853 resulted in reduced CA-IX^+^ staining in comparison with control or IMGN853 monotherapy, as shown by fluorescence intensity quantification (Fig. 5F).

**Figure 6:**
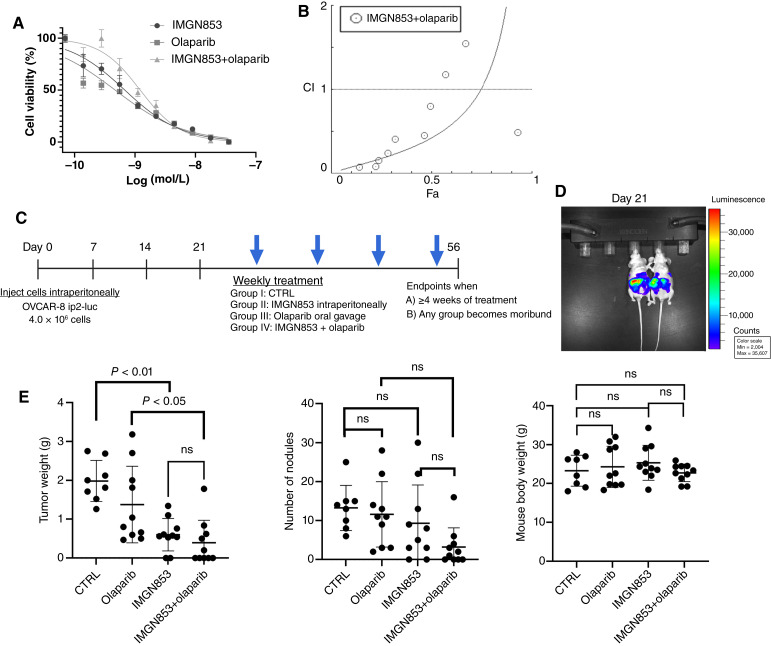
Combination therapy with IMGN853 and the PARP inhibitor olaparib in the OVCAR-8 model. **A,** MTT assay results showing the combinatorial effects of different concentrations of IMGN853+olaparib on the viability of OVCAR-8 cells after 72 hours of treatment [shown as log IMGN853(M)]. *n* = 3; data are mean ± SD. **B,** The CI plot for IMGN853 and olaparib. **C,** Schematic representation of the mouse models established using OVCAR-8 ip2-luc cells (4 × 10^6^/mouse). **D,** Twenty-one days after cell injection as shown by bioluminescence signals in the representative mice from IVIS imaging, mice were randomized to receive buffer (control), IMGN853 alone (5.2 mg/kg, once weekly), olaparib alone (50 μg/mouse/day, oral gavage), or IMGN853+olaparib. Two mice from the control group became moribund before receiving the fourth dose and were excluded from the final analysis. **E,** Tumor weights (left), numbers of tumor nodules (middle), and body weights (right) of the mice at necropsy. *P* values were determined by a two-tailed, nonparametric *t* test. Error bars ± SDs. CTRL, control; ns, not significant.

Further investigations using OVCAR-8 cells assessed the IC_50_ value of IMGN853 (Supplementary Fig. S6A) and the inhibitory effects of combinations with cisplatin (Supplementary Fig. S6B), doxorubicin (Supplementary Fig. S6C), and olaparib (Fig. 6A). Of these agents, only olaparib demonstrated a mild synergistic effect with IMGN853 (Fig. 6A). The CI (CI/Fa) for IMGN853 and olaparib was 0.83/0.4 (Fig. 6B). We examined the therapeutic efficacy of IMGN853 and olaparib, individually and in combination, using the orthotopic OVCAR-8 ip2-luc mouse model (Fig. 6C) following tumor establishment confirmed through BLI (Fig. 6D). The tumor weights and the number of tumor nodules did not differ significantly between IMGN853 alone–treated and IMGN853+olaparib-treated mice (tumor weight: *P* = 0.05; number of tumor nodules: *P* > 0.05). However, the combination treatment resulted in significantly lower tumor weights compared with olaparib alone [*P* < 0.05; Fig. 6E (left)], although the reduction in nodule numbers did not reach significance [Fig. 6E (middle)]. IMGN853 alone or in combination with olaparib was well tolerated by mice, as evidenced by similar body weights across treatment groups [Fig. 6E (right)].

To explore the role of beclin-1 in IMGN853-induced autophagic cell death, we determined cell viability using SYTOX via flow cytometry in OVCAR-8 cells and found that shRBCN1 knockdown significantly reduced the cytotoxicity induced by IMGN853 monotherapy (Supplementary Fig. S7). Compared with control or olaparib alone, both IMGN853 alone and in combination with olaparib increased cleaved caspase-3 levels, as demonstrated by IF staining (Supplementary Fig. S8A) and fluorescence intensity quantification (Supplementary Fig. S8B).

**Figure 7:**
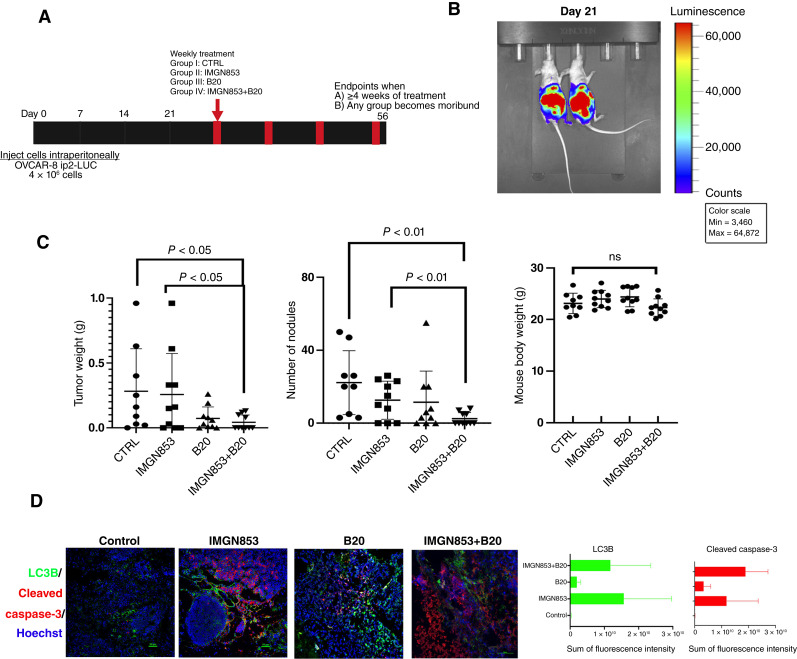
Combination therapy with IMGN853 and the anti–VEGF-A antibody B20 in the OVCAR-8 model. **A,** Schematic representation of the mouse models established using OVCAR-8 ip2-luc cells (4 × 10^6^/mouse). **B,** Twenty-one days after cell injection as shown by bioluminescence signals in the representative mice from IVIS imaging, mice were randomized to receive buffer (control), IMGN853 alone (5.2 mg/kg, once weekly), B20 alone (5 mg/kg, twice weekly, intraperitoneally), or IMGN853+B20. **C,** Tumor weights (left), numbers of tumor nodules (middle), and body weights (right) of the mice at necropsy. *P* values were determined by a two-tailed, nonparametric *t* test. Error bars ± SDs. **D,** Left, Representative IF images of tumor tissue sections from each treatment group showing the expression of the autophagy marker LC3B and the cell death marker cleaved caspase-3. Right, IF quantification was performed as described for Figure 5E. CTRL, control; ns, not significant.

Next, we investigated the effects of combining IMGN853 with antiangiogenic agents by evaluating the efficacy of treating mice bearing the orthotopic OVCAR-8 ip2-luc model with IMGN853 and the anti–human/mouse VEGF-A antibody B20, both individually and in combination (Fig. 7A), following tumor establishment confirmed through BLI (Fig. 7B). This analysis employed the same control and IMGN853 treatment groups as those used for assessing the IMGN853 and topotecan combination (Fig. 5E) as these experiments were conducted concurrently. After 4 weeks of treatment, mice treated with the IMGN853+B20 combination exhibited significantly lower tumor weights (*P* < 0.05) and fewer tumor nodules (*P* < 0.01) compared with control mice and those receiving IMGN853 alone (Fig. 7C). The combination treatment was well tolerated, with comparable body weights observed across all groups at the study’s conclusion (Fig. 7B). We further assessed expression levels of LC3B and cleaved caspase-3 as factors involved in autophagic cell death in tumor sections from each treatment group. IF staining and quantification of fluorescence intensity demonstrated that tumors treated with IMGN853 alone or in combination with B20 had elevated expression of LC3B and cleaved caspase-3 in comparison with control or B20-treated tumors (Fig. 7D). Collectively, these results suggest that combining IMGN853 with topotecan or an anti–VEGF-A antibody can significantly enhance its therapeutic efficacy by increasing autophagic cell death.

## Discussion

Understanding the heterogeneity of FOLR1 expression in ovarian tumors is crucial for selecting effective FOLR1-targeting therapeutics. Although IMGN853 has shown a significant overall survival benefit in patients with PROC who exhibit FOLR1^high^ expression, variability in FOLR1 expression may contribute to the development of adaptive drug resistance (9). Our single-cell analysis indicates that the majority of FOLR1 expression is localized in epithelial cancer cells compared with other stromal components in tumor samples from patients with HGSC. Furthermore, we found that IMGN853 induces autophagic cell death in addition to the direct effects of its cytotoxic agent. Further work on the contribution of IMGN853-induced autophagic cell death to the bystander’s killing effect within the tumor microenvironment will be informative to understand its contribution in tumor inhibition.

Furthermore, we evaluated the efficacy of combining IMGN853 with chemotherapeutics such as topotecan and olaparib, as well as the anti–VEGF-A antibody B20, and observed varied effects. Previous *in vivo* studies demonstrated that IMGN853, in combination with carboplatin, PEGylated liposomal doxorubicin, or bevacizumab, exhibited antitumor activity in an OV-90 subcutaneous xenograft model (29). Subsequent clinical trials combining IMGN853 with bevacizumab reported an objective response rate of 39% (30). In our therapeutic studies using orthotopic HGSC models, IMGN853 showed greater efficacy when combined with topotecan or B20 compared with IMGN853 monotherapy. On the other hand, our results indicated that the combination of IMGN853 with B20 did not result in a significant difference in comparison with B20 monotherapy in tumor weight reduction. Whether such combinations could be useful in tumors that are resistant to anti-VEGF antibody drugs is not known.

Cancer cells are highly dependent on FOLR1 internalization to initiate folate metabolism for DNA replication, methylation, and repair (31). Our observations of increased autophagic cell death, marked by elevated LC3B and activated caspase-3 levels following IMGN853 treatment in HGSC cell lines and orthotopic tumors, highlight the importance of FOLR1-mediated autophagic cell death in the inhibitory effects of IMGN853. Further research is required to determine whether IMGN853-induced autophagic cell death is modulated by hypoxia and angiogenesis, providing a more comprehensive understanding of the indirect effects of IMGN853 and their contribution to tumor growth inhibition.

Our study acknowledges several limitations. The variability in tumor uptake rates in the PARP inhibitor (olaparib) group prevented full assessment of its combination effects. Further studies are needed to determine whether combining IMGN853 with other platinum-based agents, such as carboplatin, could result in producing synergistic effects in FOLR1^high^ ovarian cancer. Long-term survival experiment and/or a tumor regression experiment on each combination strategy may also be helpful. All mouse groups in the combination studies displayed minimal toxicity, as evidenced by stable body weights, consistent with the safety profile reported in a phase I expansion study (32). Additional studies on how IMGN853-induced autophagy facilitates cell death under chemotherapeutic stress from topotecan or an antiangiogenic agent are needed to improve our understanding of the role of autophagic cell death in these tumor growth–inhibitory effects of IMGN853.

In this study, we used an array of orthotopic HGSC models including platinum-resistant HGSC PDX and cell-based models to test the inhibitory efficacy of IMGN853 alone or in combination with chemotherapeutic drugs. Although our single-cell analysis showed predominant expression of FOLR1 in epithelial cancer cells, our results were potentially constrained by the heterogeneous FOLR1 expression in HGSC tumors. Additionally, our studies could be bolstered by employing syngeneic models with immunocompetent tumor microenvironments to assess how IMGN853 affects and is affected by the immune microenvironment. Studies focusing on immune components within the tumor microenvironment could clarify FOLR1’s role in mediating interactions between cancer cells and immune compartments, providing valuable insights for selecting IMGN853 and immunotherapy combinations.

Collectively, our findings suggest promising IMGN853 combination therapies for clinical application, such as combining IMGN853 with topotecan or an anti–VEGF-A antibody. These findings, coupled with our mechanistic insights on IMGN853-induced autophagic cell death, thus provide a strong foundation for advancing the clinical development of IMGN853 in treating ovarian cancer.

## Supplementary Material

Supplementary Figures Supplementary Figures S1 to S8

Supplementary Table 1 Expression of FOLR1 in ovarian cancer patients according to patient characteristics including age, disease stage, and site (primary or metastatic) of sampled tumor

Supplementary Table 2 Supplemental Table 2. Antibodies, critical chemical assays and other related materials/software

Supplementary Table 3 Supplementary Table S3. Ovarian cancer cell lines’ FOLR1 expression levels extracted from the DepMap Public 22Q4 Database. The cut-off of FOLR1 Expression log2(TPM+1) is 2.0.

Supplementary Movie 1 Time-lapse videos of GFP and RFP fluorescence in ptLC3-pEGFP-RFP–expressing OVCAR-8 cells during treatment with the control formulation buffer (Movie 1)

Supplementary Movie 2 Time-lapse videos of GFP and RFP fluorescence in ptLC3-pEGFP-RFP–expressing OVCAR-8 cells during treatment with IMGN853 (Movie 2)
