# Supplementary material for: IMGN853 Induces Autophagic Cell Death in Combination Therapy for Ovarian Cancer
Source: Cancer Res Commun. 2025 Mar 28;5(3):512–26. doi: 10.1158/2767-9764.CRC-24-0215 (PMC11951858; doi:10.1158/2767-9764.CRC-24-0215)
Supplement: Supplementary Figures — S1 to S8 [file crc-24-0215_figure_suppsf1_to_f8.pdf]

Supplementary Figure 1

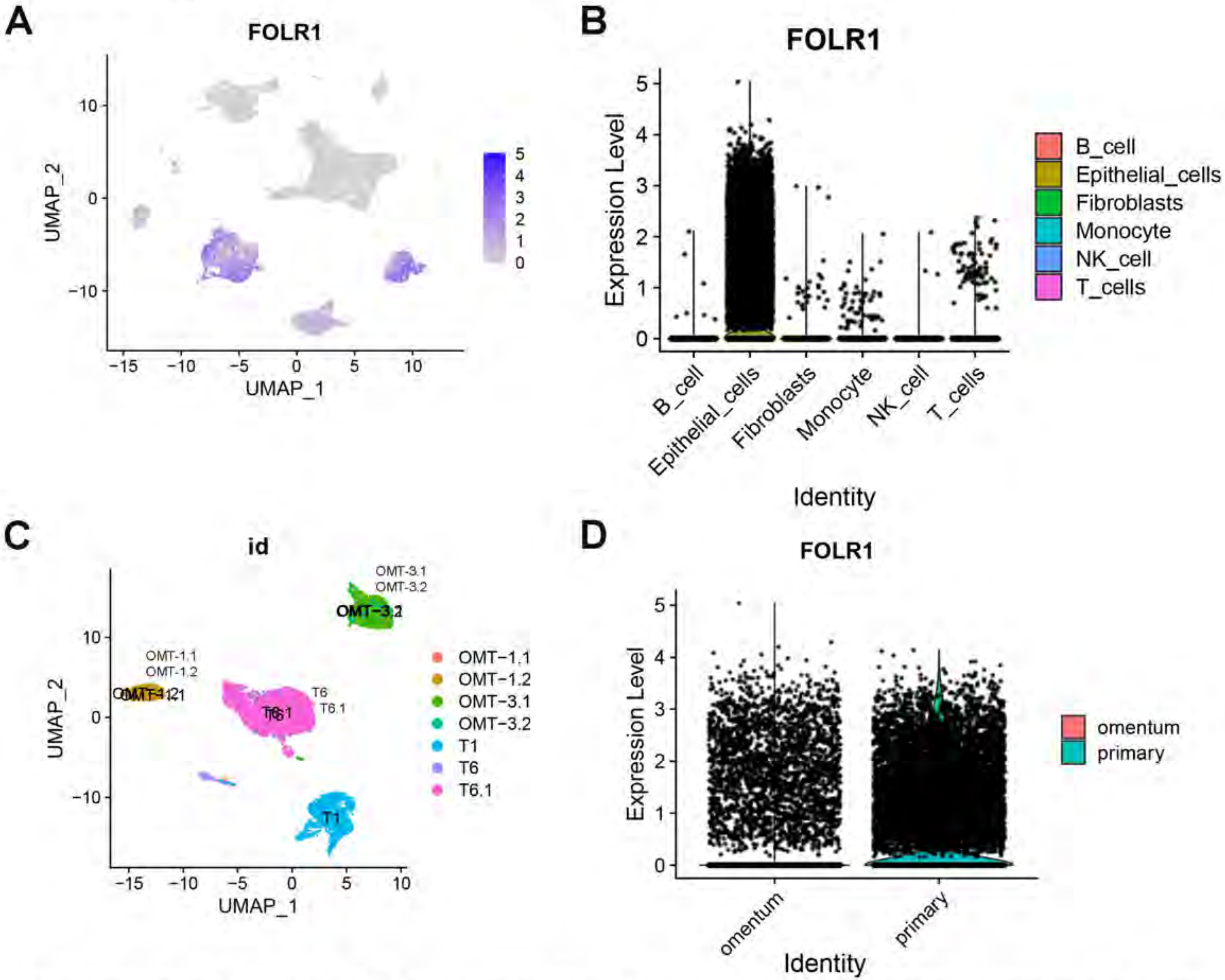

Supplementary Figure S1. FOLR1 is predominantly expressed in epithelial ovarian cancer cells. **(A and B)** Single-cell analysis of FOLR1 expression visualized by UMAP (A) and violin plots (B) in different cell types. **(C)** UMAP visualization of primary tumor and omental metastatic epithelial high-grade serous ovarian cancer (HGSC) cells. OMT, omentum; T, primary tumor. **(D)** Violin plots show FOLR1 expression levels clustered by sample origin (omentum or primary tumor).

Supplementary Figure 2

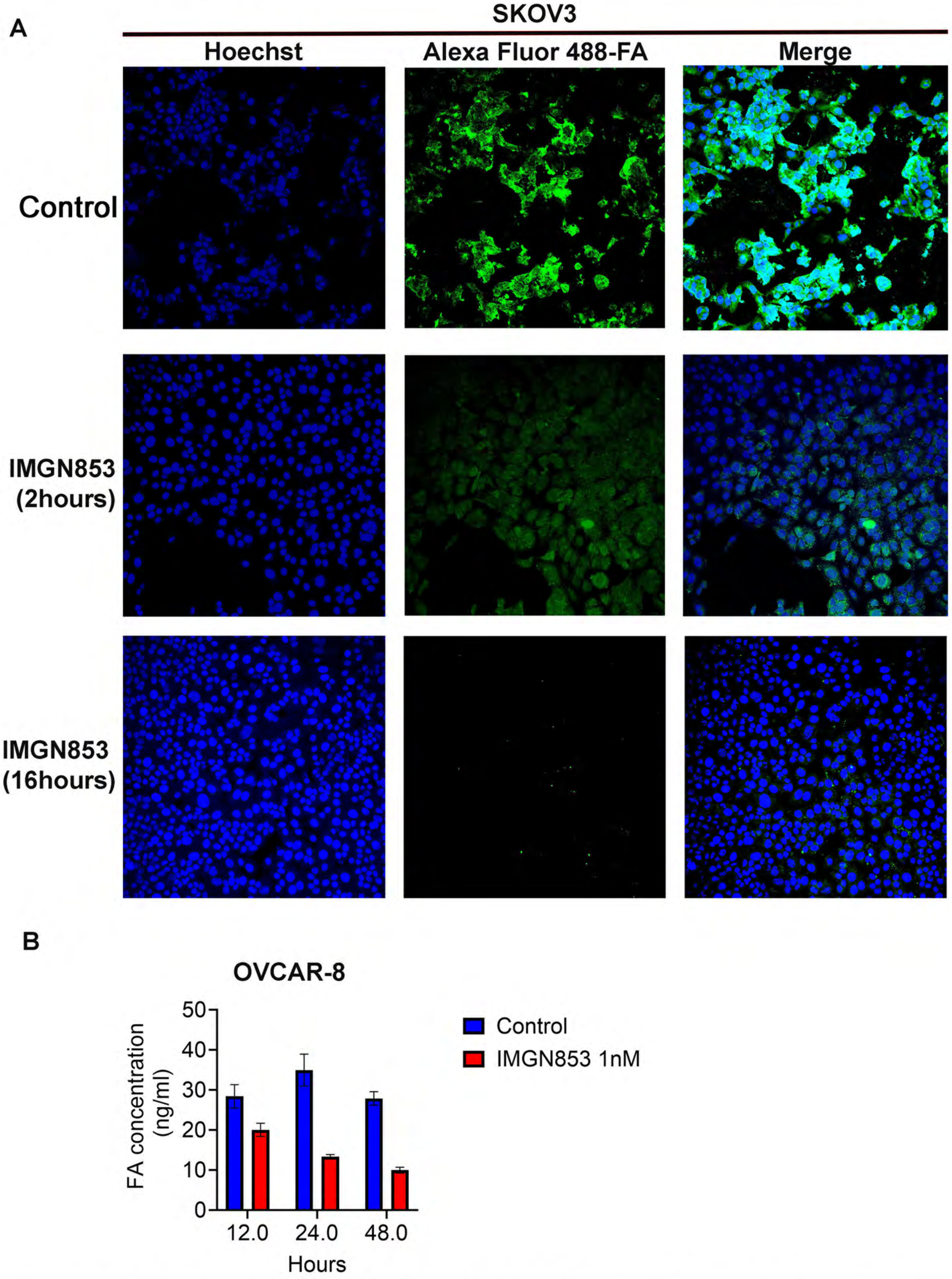

Supplementary Figure S2. IMGN853 blocks folic acid (FA) uptake and the intracellular FA levels in ovarian cancer cells. (A) Representative immunofluorescence images show that, compared with cells with no pretreatment (CTL), SKOV3 cells pretreated with IMGN853 for 2 h have decreased FA uptake, whereas those pretreated with IMGN853 for 16 h have no FA uptake. (B) Intracellular FA was quantitatively measured by colorimetric FA ELISA kit in protein lysates from OVCAR-8 cells with no-pretreatment (CTL) or IMGN853 at 12hrs, 24 hrs and 48 hrs time point, $n = 3$.

Supplementary Figure 3

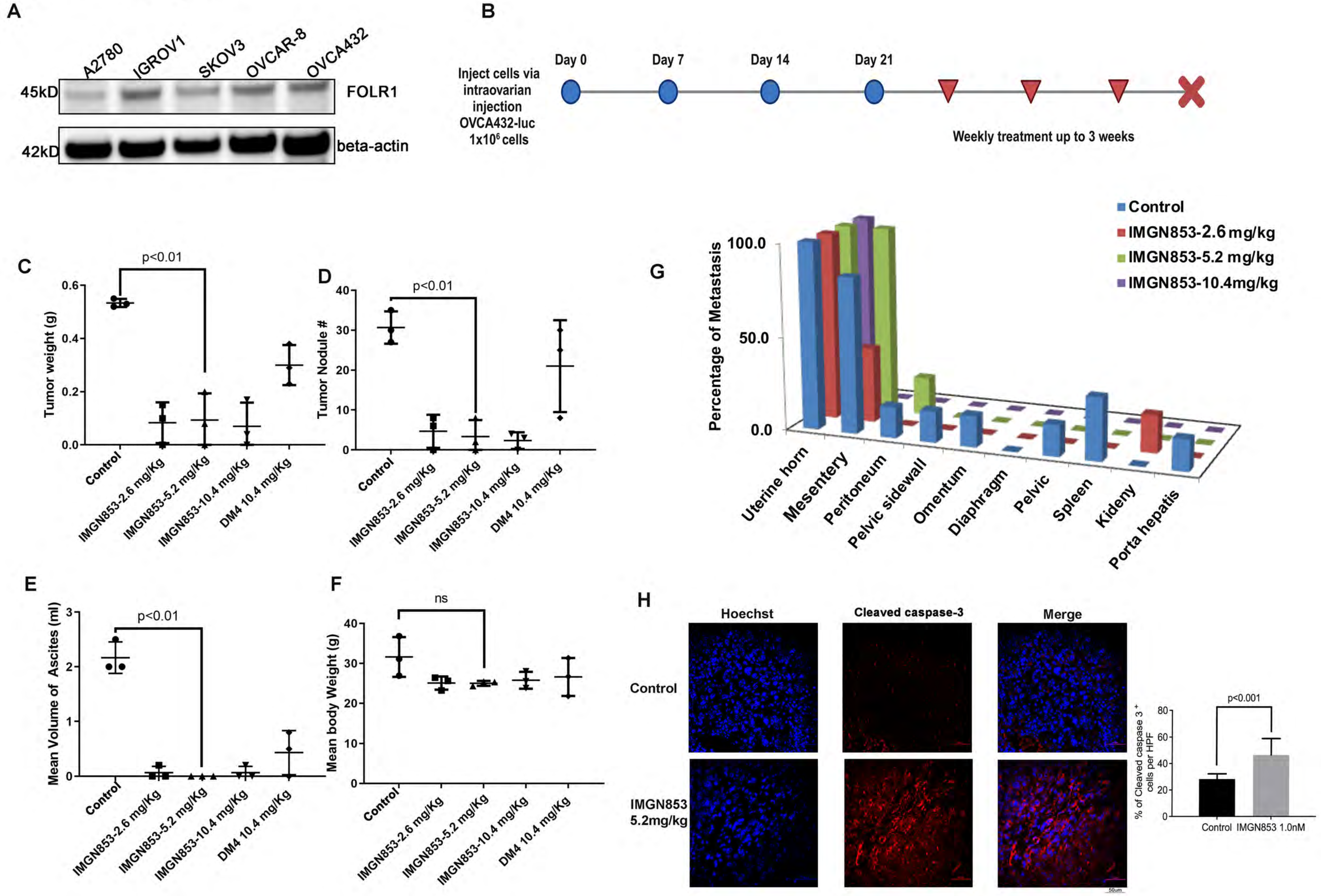

Supplementary Figure S3. *In vivo* dose-finding experiments in ovarian cancer models through surgical orthotopic injection. (A) Protein expression of FOLR1 in ovarian cancer cells (endometrioid ovarian cancer cells SKOV3, IGROV1, A2780, and high-grade serous ovarian cancer (HGSC) cell lines (OVCA432 and OVCAR-8). β-actin was applied as the loading control. **(B)** Schematic representation of IMGN853 dose-finding experiment in orthotopic FOLR1⁺ HGSC model OVCA432. Orthotopic tumors were established from OVCA432 cells (1 × 10^6/mouse i.p. injection). Three mice were assigned in each group for this experiment. Twenty-one days after cell implantation, mice were randomized to receive control (formulation buffer); 2.6 mg/kg, 5.2 mg/kg, or 10.4 mg/kg IMGN853; or 10.4 mg/kg Ch-KTI-s-SPDB-DM4 (DM4, nonbinding antibody control). **(C-F)** Tumor weights (C), numbers of tumor nodules (D), volumes of ascites (E), and body weights (F) of the mice at necropsy. Tumor weight: control vs. 5.2 mg/kg IMGN853, $P < 0.01$; number of nodules: control vs. 5.2 mg/kg IMGN853, $P < 0.01$; mean volume of ascites: control vs. 5.2 mg/kg IMGN853, $P < 0.01$. ns, not significant. **(G)** Percentage of distribution of metastatic nodules at the indicated anatomic sites in mice treated with control buffer (CTL) or 2.6 mg/kg (0.5 nM), 5.2 mg/kg (1.0 nM), or 10.4 mg/kg (2.0 nM) IMGN853. **(H) Left:** Representative immunofluorescence images show the expression of cleaved caspase 3 in tumors from mice treated with control buffer or IMGN853 (5.2 mg/kg). Scale bar = 50 μm. **Right:** Quantification of the percentage of cells expressing cleaved caspase 3 per high-power field. n=3; error bars represent SEM.

Supplementary Figure 4

A

Antibody	Folate Receptor RxDX	Vendor	Ventana
Clone	FOLR1-2.1	Catalog	740-5065
Retrieval	Ultra CC1	Control	Fallopian tube: Eptithelium positive control. Lamina propria and smooth muscle negative control.
Dilution	Pre-dilute, Ready to Use	Platform	BenchMark ULTRA
Lot	J33097	Detection	Optiview DAB IHC Detection

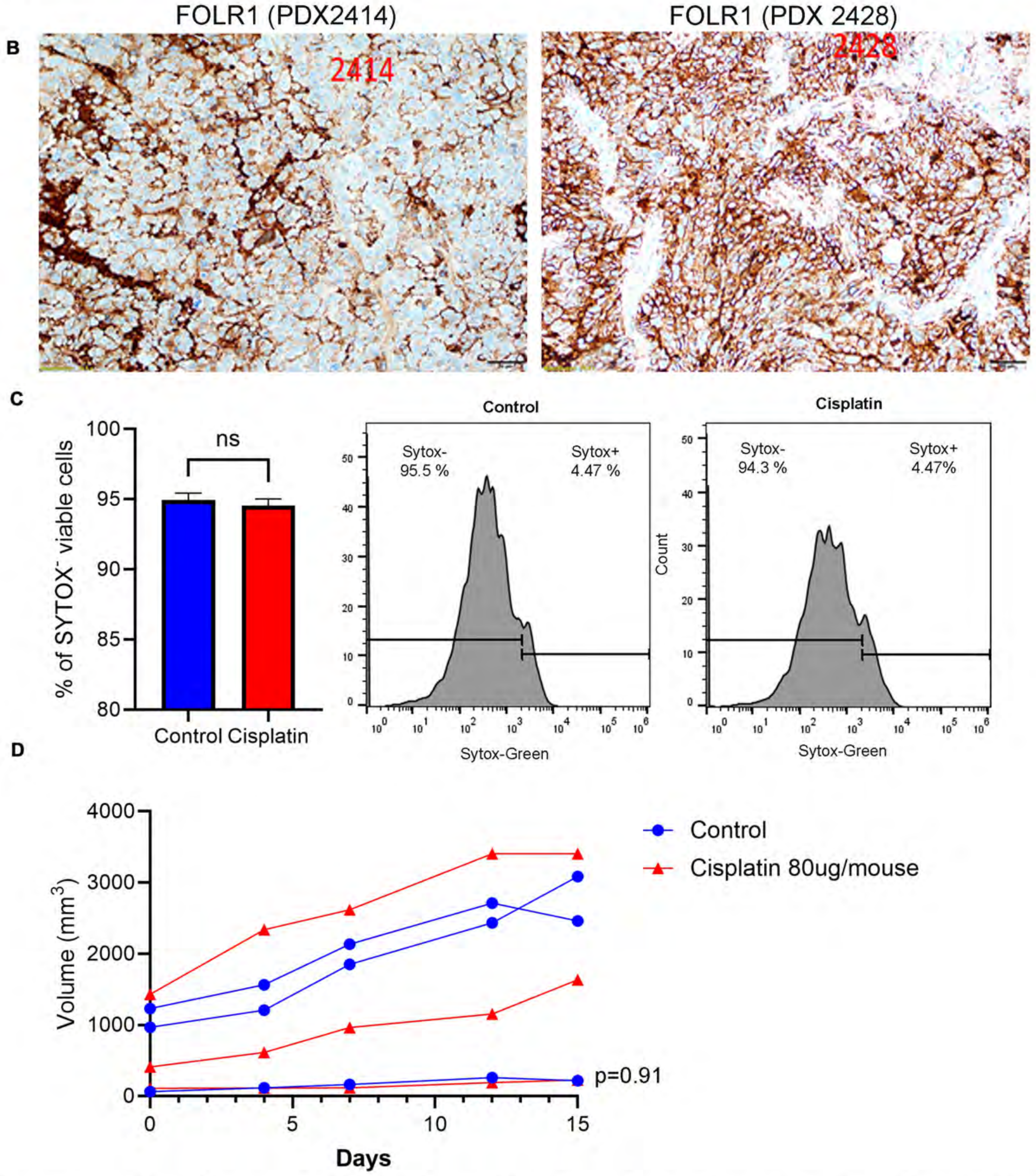

Supplementary Figure S4. Pathological and clinical characteristics of patients from whom PDX tumors were established. (A) Conditions of immunohistochemical (IHC) staining; **(B)** Representative IHC images for expression of FOLR1 in HGSC PDX 2414 and 2428 tumors. **(C)** Right: HGSC PDX tumor cells were harvested from the initial subcutaneously implanted tumors by mechanical dissociation and recycled, then the attached tumor cells were treated with control or cisplatin 2uM for 72 hrs, *n* = 3; data are mean ± SD. Left: Representative plots for SYTOX-FITC flow cytometry analysis. **(D)** Subcutaneously implanted HGSC PDX 2428 tumors were treated with either control or cisplatin (4 mg/kg, i.p. once weekly) for approximately 2 weeks. Tumor volumes (V) were calculated by V=0.5 x Length x Width^2; Statistical analysis was two-way ANOVA, Overall effect of treatment: p = 0.91 (Interaction: p = 0.98).

Supplementary Figure S5

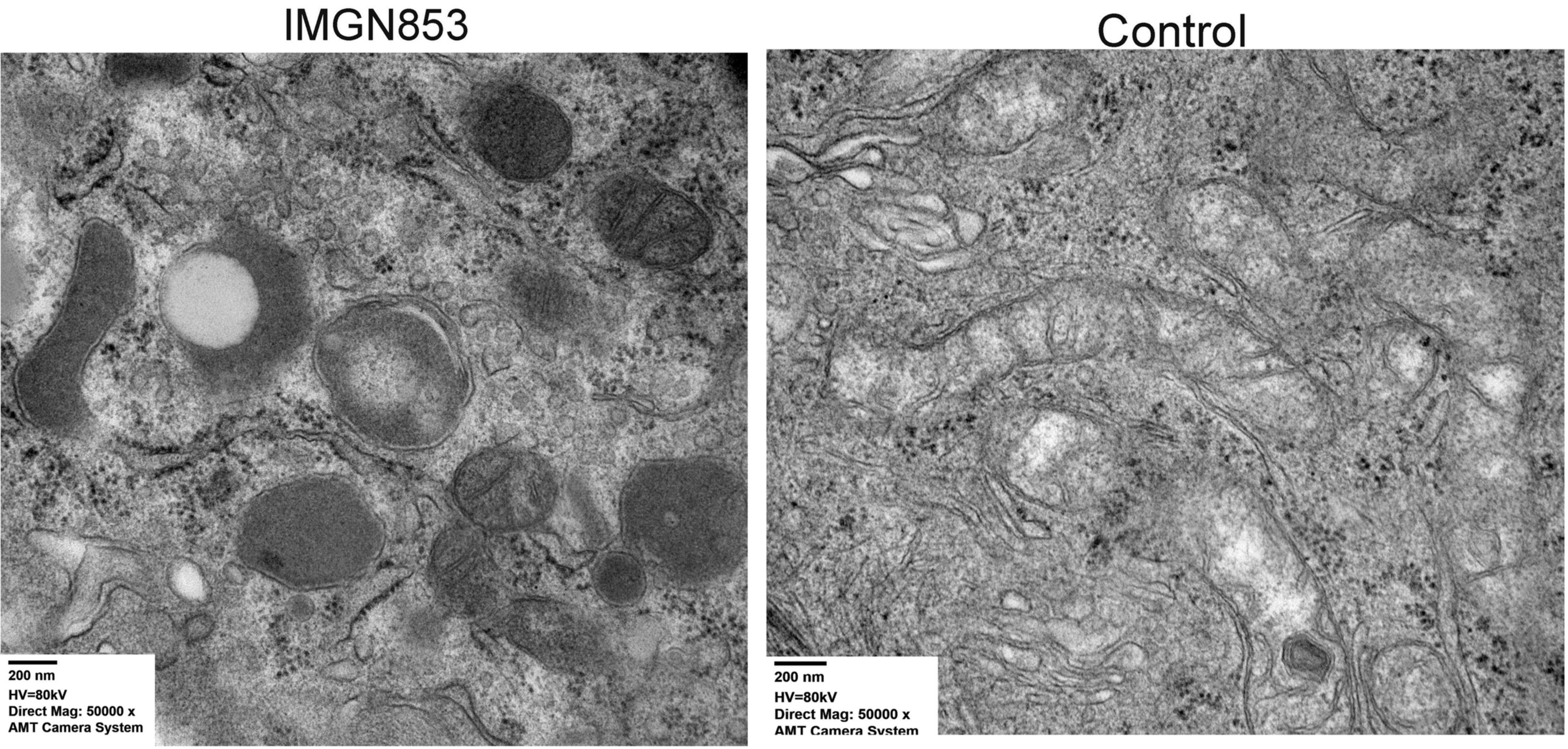

Supplementary Figure S5. High-resolution images from transmission electron microscopy (TEM). Additional pair of OVCAR-8 tumors treated with (Left panel) IMGN853 5.2 mg/kg (1.0 nM), i.p. per week or (Right panel) control buffer for 6-week doses OVCAR-8 tumors treated were shown autophagic vacuoles at magnifications 50,000×. A pair of different tumor samples from Figure 3C were shown, and representative images were selected from 6 individual repeats in each treatment.

Supplementary Figure 6

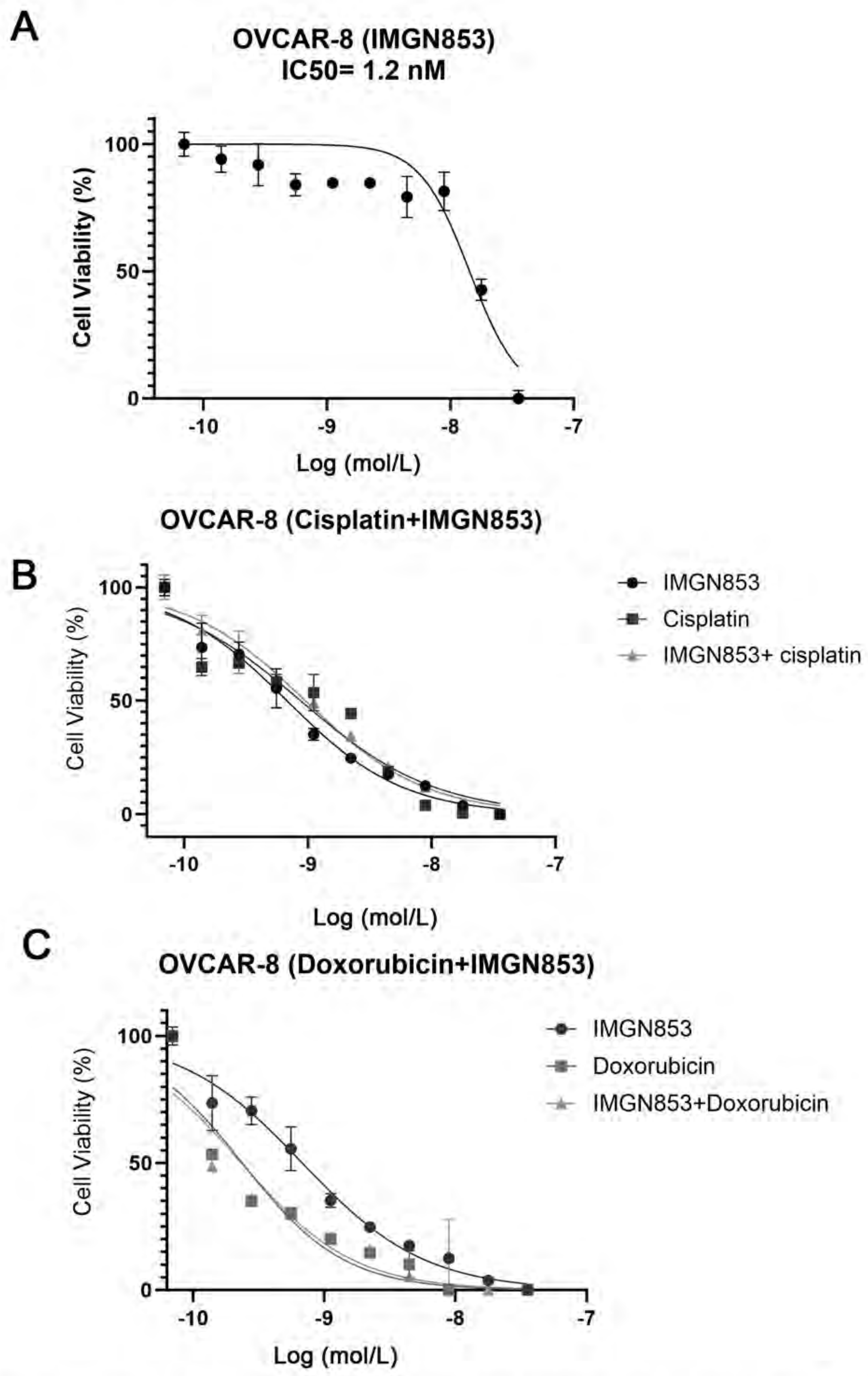

Supplementary Figure S6. *In vitro* cytotoxicity of combinations of IMGN853 with conventional chemotherapeutics. (A) Viability of OVCAR-8 cells 72 h after treatment with various concentrations of IMGN853. In OVCAR-8 cells, the IC_{50} of IMGN853 was approximately 1.2 nmol/L. **(B)** Viability of OVCAR-8 cells 72 h after treatment with various concentrations of IMGN853 or cisplatin alone or in combination. $n = 3$; data are means ± SDs. **(C)** Viability of OVCAR-8 cells 72 h after treatment with various concentrations of IMGN853 or doxorubicin alone or in combination. $n = 3$; data are mean ± SD.

Supplementary Figure 7

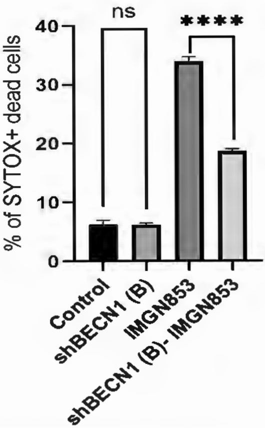

Supplementary Figure S7. Role of beclin1 in cytotoxicity of IMGN853 induced autophagic cell death. Viability of OVCAR-8-WT or -shBECN1 (B) knockdown cells treated with IMGN853 for 72 h and analyzed by SYTOX-orange-stained flow cytometry. *P* values were determined by a two-tailed, nonparametric *t*-test. *n* = 3; data are mean ± SD.

Supplementary Figure 8

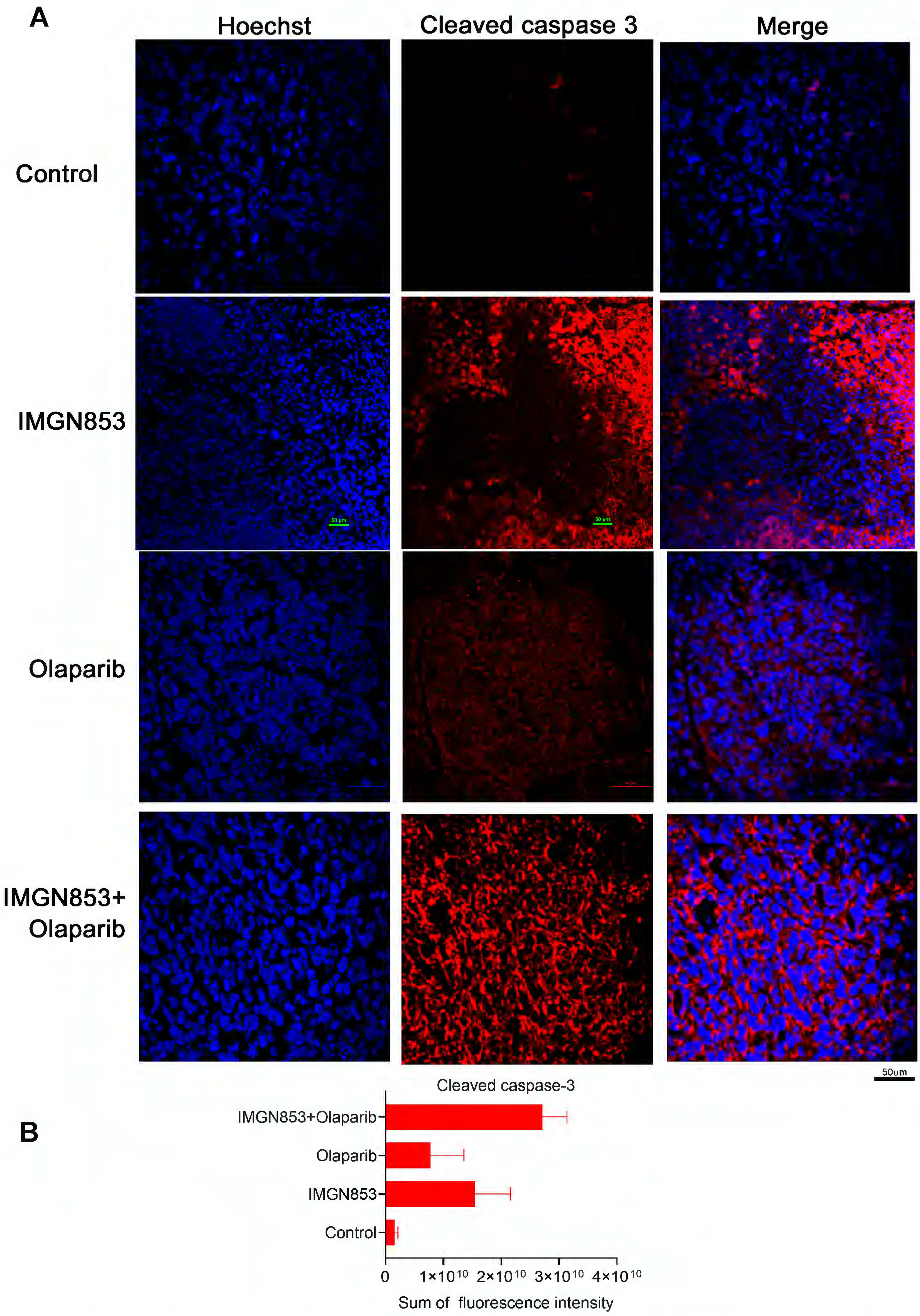

Supplementary Figure S8. Tumors treated with IMGN853 alone or in combination with olaparib have increased cleaved caspase 3 expression. (A) Representative immunofluorescence images of tumor sections stained for cleaved caspase 3 (red) from mice treated with control, IMGN853 (5.2 mg/kg), olaparib (50 μg/mouse/day), or IMGN853+olaparib. Scale bar = 50 μm. (B) Immunofluorescence quantification was performed as described for Fig. 5E.
