# Supplementary material for: IMGN853 Induces Autophagic Cell Death in Combination Therapy for Ovarian Cancer
Source: Cancer Res Commun. 2025 Mar 28;5(3):512–26. doi: 10.1158/2767-9764.CRC-24-0215 (PMC11951858; doi:10.1158/2767-9764.CRC-24-0215)
Supplement: Supplementary Table 2 — Supplemental Table 2. Antibodies, critical chemical assays and other related materials/software [file crc-24-0215_supplementary_table_2_suppst2.docx]

**Supplemental Table 2. Antibodies, critical chemical assays and other related materials/software**

| **Reagent or resource** | **Source** | **Identifier** |
| --- | --- | --- |
| **Experimental models: cell lines** | | |
| A2780 | MDA Cell Line Core | CVCL_0134 |
| SKOV3 | MDA Cell Line Core | CVCL_0532 |
| IGROV-1 | MDA Cell Line Core | CVCL_1304 |
| OVCAR-8 | MDA Cell Line Core | CVCL_1629 |
| OVCA432 | MDA Cell Line Core | CVCL_3769 |
| **Experimental models: organisms/strains** | | |
| Female athymic nude mice (NCr-nu) | Taconic (10 mice/group) | N/A |
| **Chemicals, peptides, and recombinant proteins** | | |
| Optimal cutting temperature (OCT) media | Miles, Inc. | Catalog no. 25608-930 |
| Lipofectamine 2000 | Invitrogen | Catalog no. 11668027 |
| Topotecan | Sigma-Aldrich | 123948-87-8 |
| olaparib | Tecoland | 763113-22-0 |
| Acridine Orange | Sigma | A8097-10ML |
| Hydroxychloroquine | Sigma | H0915-5MG |
| B20 | Genentech, South San Francisco, CA | N/A |
| MTT reagent | Sigma-Aldrich | Catalog no. M2128 |
| DM4 | N2’-[4-[(3-carboxypropyl)dithio]-4-methyl-1-oxo-2-sulfopentyl]-N2’-  deacetylmaytansine | Immunogen |
| **Antibodies: Expressed in HEK293 suspension cells and purified with use of protein A affinity resin to >95% purity.** | | |
| FOLR1 (polyclonal rabbit anti‑human FOLR1/folate receptor alpha antibody) | LSBio | Catalog no. LS‑C818010  RRID:# NA |
| FOLR1 (polyclonal folate receptor alpha antibody) | Thermo Fisher | Catalog no. PA5-24186  RRID:AB_2541686 |
| Beclin-1 | Cell Signaling Technology | Catalog no. CST-3738S, RRID:AB_490837 |
| HIF1-alpha polyclonal antibody | Thermo Fisher Scientific | Cat# PA1-16601, RRID:AB_2117128 |
| CD31 | Pharmingen | Catalog no. 557355, RRID:AB_396660 |
| LC3B (Polyclonal rabbit anti-human and anti-mouse antibody) | Cell Signaling Technology | Catalog no. 2775, RRID:AB_915950 |
| β-actin | Sigma-Aldrich | Catalog no. A5441, RRID:AB_476744 |
| Vinculin | Sigma-Aldrich | Catalog no. V9131, RRID:AB_477629 |
| Anti-rabbit secondary antibodies conjugated with horseradish peroxidase | Sigma-Aldrich | Catalog no. NA934, RRID:AB_772206 |
| Anti-mouse secondary antibodies conjugated with horseradish peroxidase. | Sigma-Aldrich | Catalog no. NA931, RRID:AB_772210 |
| pLenti-C-mGFP-human CD5L vector | OriGene | Catalog no. RC206528L2  RRID:# NA |
| **Critical commercial assays** | | |
| SYTOX Blue Dead Cell Stain | Thermo Fisher | Catalog number: S34857 |
| **Software and algorithms** | | |
| GraphPad Prism 7.0 | GraphPad Software | RRID:SCR_002798 |
| SPSS 12 for Windows | SPSS, Inc. |  |
| R statistical package 3.4.1 | R Foundation for Statistical Computing |  |
| **Software and algorithms** | | |
| Windows statistical software | SPSS version 12 for Windows statistical software | SPSS, Inc., Chicago, IL |
| R statistical package | R.3.4.1 | R Foundation for Statistical Computing, Vienna, Austria |
| ImageJ | ImageJ 1.52a | Wayne Rasband, National Institutes of Health, Bethesda, MD |
| Clustal3.0 software | http://bonsai.hgc.jp/~mdehoon/software/cluster/software.htm | de Hoon M.J.  Imoto S.  Nolan J.  Miyano S.  Open source clustering software.  Bioinformatics. 2004; 20: 1453-1454 |
| TreeViewX program | http://jtreeview.sourceforge.net/ | Saldanha A.J.  Java Treeview--extensible visualization of microarray data.  Bioinformatics. 2004; 20: 3246-3248 |
| CompuSyn | www.combosyn.com |  |
