# Supplementary material for: IMGN853 Induces Autophagic Cell Death in Combination Therapy for Ovarian Cancer
Source: Cancer Res Commun. 2025 Mar 28;5(3):512–26. doi: 10.1158/2767-9764.CRC-24-0215 (PMC11951858; doi:10.1158/2767-9764.CRC-24-0215)
Supplement: Supplementary Table 3 — Supplementary Table S3. Ovarian cancer cell lines’ FOLR1 expression levels extracted from the DepMap Public 22Q4 Database. The cut-off of FOLR1 Expression log2(TPM+1) is 2.0. [file crc-24-0215_supplementary_table_3_suppst3.docx]

**Supplementary Table S3. Ovarian cancer cell lines’ FOLR1 expression levels extracted from the DepMap Public 22Q4 Database.** The cut-off of FOLR1 Expression log2(TPM+1) is 2.0.

| **Cell Line** | **Type** | **Primary Disease** | **FOLR1 Expression**  **log2 [TPM+1] Expression Public 24Q2** |
| --- | --- | --- | --- |
| OVCAR8 | HGSC | Ovarian Epithelial Tumor | 3.698218478 |
| OAW28 | HGSC | Ovarian Epithelial Tumor | 7.489446599 |
| KURAMOCHI | HGSC | Ovarian Epithelial Tumor | 3.702657543 |
| NIHOVCAR3 | HGSC | Ovarian Epithelial Tumor | 4.341985747 |
| OVMANA | HGSC | Ovarian Epithelial Tumor | 7.638653116 |
| CAOV4 | HGSC | Ovarian Epithelial Tumor | 5.135863165 |
| CAOV3 | HGSC | Ovarian Epithelial Tumor | 5.768184325 |
| SNU8 (40) | HGSC | Ovarian Epithelial Tumor | 2.659924558 |
| OVTOKO | HGSC | Ovarian Epithelial Tumor | 1.316145742 |
| JHOS2 | HGSC | Ovarian Epithelial Tumor | 8.59484709 |
| JHOS4 | HGSC | Ovarian Epithelial Tumor | 7.781687562 |
| **Cell Line** | **Type** | **Primary Disease** | **FOLR1 Expression**  **log2 [TPM+1] Expression Public 24Q2** |
| SKOV3 | Non-HGSC | Ovarian Epithelial Tumor | 6.397802962 |
| IGROV1 | Non-HGSC | Ovarian Epithelial Tumor | 9.069986734 |
| A2780 | Non-HGSC | Ovarian Epithelial Tumor | 1.310340121 |
